# Extrapolation of PBBs Environmental Transformation Mechanisms and Toxicity Risks of Byproducts

**DOI:** 10.3390/ijms26041753

**Published:** 2025-02-19

**Authors:** Bohan Xu, Qian Liu, Weihan Cui, Li Tao, Yuanquan Chi, Luze Yang

**Affiliations:** 1 College of Resources and Environment, Jilin Agricultural University, Changchun 130118, China; xbh2275850139@163.com (B.X.); 19274302086@163.com (W.C.); q3364367547@163.com (L.T.); sourcechi6@163.com (Y.C.); 2 State Key Laboratory of Environmental Criteria and Risk Assessment, Chinese Research Academy of Environmental Sciences, Beijing 100012, China; liuqiankaoyan@163.com; 3 National Engineering Laboratory for Lake Pollution Control and Ecological Restoration, Chinese Research Academy of Environmental Sciences, Beijing 100012, China

**Keywords:** polybrominated biphenyls, transformation mechanisms, TOPKAT model, human toxicities, risk evaluation

## Abstract

Polybrominated biphenyls (PBBs) are commonly used flame retardants that pose severe risks to humans. However, there is a lack of systematic research on the transformation process and biological toxicities of PBBs in the environment, which is not conducive to the prevention and control of pollution risks of PBBs. Therefore, the transformation pathways (i.e., photodegradation, microbial degradation, combustion oxidation, and in vivo metabolism) of PBBs and previously designed PBB substitutes were deduced first. Then the potential rodent carcinogenicity, rodent toxicity, mutagenicity, developmental toxicity, skin and eye irritation, skin sensitization, and aquatic toxicity of the transformation products were evaluated using the toxicokinetics (TOPKAT) model. Finally, 3D quantitative structure activity relationship (3D-QSAR) models were constructed to assess the human toxicity (i.e., carcinogenicity, developmental toxicity, hepatotoxicity, epigenetic toxicity, neurotoxicity, and immunotoxicity) of PBBs, PBBs substitutes, and their transformation products. Results showed that the transformation products of PBBs and their substitutes exhibit high toxicity risks (i.e., potential carcinogenicity, mutagenicity, and developmental toxicity) to organisms. The D3-A1 molecule had the highest carcinogenic risk probability at 0.826. The dihydroxy metabolite 2,2′-OH-PBB-80 of the PBB-80 molecule presented the highest potential developmental toxicity risk (toxicity probability 0.713). Polybrominated dibenzofuran (PBDF) showed the strongest skin irritation (probability 0.995). The combustion oxidation products of PBBs exhibited higher potential ecological and human health risks than other transformation products. Among potential toxicity risks to humans, the developmental toxicity of the transformation products of PBBs and their substitutes was theoretically significant, with characterization values ranging from 70.53 to 100.87. This is the first study to comprehensively evaluate the ecological and human health risks of PBBs and their transformation products by combining the inference of transformation pathways with the prediction of transformation product toxicities, providing theoretical support for the design of environmentally friendly PBB substitutes in future studies.

## 1. Introduction

PBBs are artificial chemicals that have been utilized as flame retardants in many commercial products since the 1970s [1]. In early 1973, the Velsicol Chemical Company accidentally sent PBBs to the Michigan Farm Bureau Service instead of magnesium oxide, a commonly used feed-grade nutrient supplement, causing severe harm to the environment and living organisms [2]. PBB exposure can cause heightened health risks. Studies have shown that PBBs are associated with hypothyroidism [3,4,5]. Infants whose mothers had detectable PBB were more likely to have below-median Apgar scores [6]. Breastfed girls exposed to high levels of PBB in utero (> or =7 parts per billion) had an earlier age at menarche (mean age = 11.6 years) than breastfed girls exposed to lower levels of PBB in utero (mean age = 12.2–12.6 years) or girls who were not breastfed (mean age = 12.7 years) [7]. Exposure to PBBs increased the risk of breast cancer [8]. Compared to those with the lowest exposure (≤1 ppb), those with mid-range (>1–3.16 ppb) and high (≥3.17 ppb) PBB exposure had increased odds of spontaneous abortion [9]. A repeat test revealed a virtually complete persistence of the immune dysfunctions in Michigan farmers exposed to PBB a decade ago [10]. A threefold increase in the incidence of hernia or hydrocele was found among the sons of women accidentally exposed to higher levels of PBBs [11]. Due to their bioconcentration, toxicity, environmental persistence, and long-range transportation, some PBBs, such as hexabromobiphenyl (PBB-153) and decabromobiphenyl (PBB-209), were prohibited in 2009 [12]. The European Union’s Restriction of Hazardous Substances (RoHS) Directive, China’s Management Measures for the Restricted Use of Hazardous Substances in Electrical and Electronic Products, Japan’s industry standard of the Marking for presence of the specific chemical substances for electrical and electronic equipment (JIS C 0950), and the United States’ Federal Electronic Device Environmental Evaluation (EDEE) Act all impose restrictions on the utilization of PBBs in electrical and electronic equipment, stipulating a maximum concentration limit of 0.1% (1000 parts per million). Despite restrictions on the use of PBBs, they can also be detected in the environment.

Existing PBBs release toxins into the environment via various pathways. Brominated POPs (including PBBs) were mainly released into the environment through uncontrolled combustion and incineration of household waste products [13]. The combustion of PBBs may form notorious PBDF, which poses a more severe risk to the environment [14]. Photochemical reactions are essential for the degradation of PBBs, with dehalogenation being the dominant mechanism [15,16]. Additionally, anaerobic microorganisms can degrade PBBs and produce debromination products [17]. PBBs can also be metabolized by P450 enzymes in living organisms. Metabolic transformation by P450 enzymes introduces polar groups, such as hydroxyl and carboxyl groups, into the molecular structure of pollutants, thereby enhancing their water solubility, detoxification, and excretion. The intermediates or products of PBBs during metabolic transformation result in more significant toxicity because they may bind more easily to biological macromolecules (e.g., proteins and nucleic acids) than their parent compounds [18]. Thus, the investigation of the risks of degradation pathways of PBBs is necessary; however, this remains unclear. Previous research on the transformation products of PBBs primarily focused on the single pathway such as photodegradation [19], microbial degradation [17], combustion oxidation [14], and metabolism [20], as well as the corresponding products. However, these studies were limited in terms of the number of molecules investigated, lacked multiple pathways, and were deficient in assessing the diverse toxicological risks associated with the transformation products, thereby exhibiting certain limitations. Therefore, it is significant to infer different transformation pathways and products of PBBs, as well as to evaluate the various toxicological risks of these transformation products.

Computer-based pathway extrapolation and computational toxicology have become practical tools for screening toxic compounds and assessing toxicity, and in some cases, can replace traditional toxicity testing methods [21,22]. Yamada et al. and Xu et al. applied theoretical methods to deduce the photodegradation fates of cis-chlordane, trans-chlordane, and heptachlor in ethanol and the pathways of marine polybrominated diphenyl ethers (PBDEs) in anaerobic degradation [23,24]. Elnaggar et al. and Zhang et al. used the TOPKAT method to evaluate the toxicological properties of Austalide substitutes from marine-derived *Aspergillus* sp. and metformin chlorination byproducts, respectively [25,26]. Jia et al. and Huang et al. constructed quantitative structure-activity relationship (QSAR) models to evaluate the toxicity of chemicals on Tetrahymena pyriformis and nitroaromatic compounds in humans, respectively [27,28]. However, these methods have never been used to address the toxicities of the PBBs transformation products in different pathways.

Therefore, this study evaluated the risks of PBBs generation via different pathways (i.e., photodegradation, microbial degradation, combustion oxidation, and metabolic transformation) using in silico approaches. PBBs transformation pathways for photodegradation, microbial degradation, combustion oxidation in environmental media, and metabolism in organisms were deduced and collated. TOPKAT and 3D-QSAR models of PBB human toxicities were then constructed and used to assess the transformation products of PBBs and their previously designed substitutes. This study developed a novel dual-focused methodology that systematically investigated transformation mechanisms of PBBs while establishing their integrated risk assessment framework, ultimately identifying critical yet overlooked environmental risks associated with PBBs transformation products. It provides a comprehensive perspective on the risks of PBBs and previously designed alternatives’ intermediates in different transformation pathways and a theoretical reference for the study of environmental risks and biological hazards of other pollutants.

## 2. Results and Discussion

### 2.1. Extrapolation of PBBs Environmental Transformation Pathways and the Summary of Their Transformation Products

PBB-153 is one of the main components of brominated flame retardants and has been frequently detected in both environmental media and organisms [29]. Therefore, in this study, PBB-153 was used as the primary example for the extrapolation of PBBs’ environmental transformation pathways.

#### 2.1.1. Extrapolation of PBBs Photolysis Pathways

**Figure 1:**
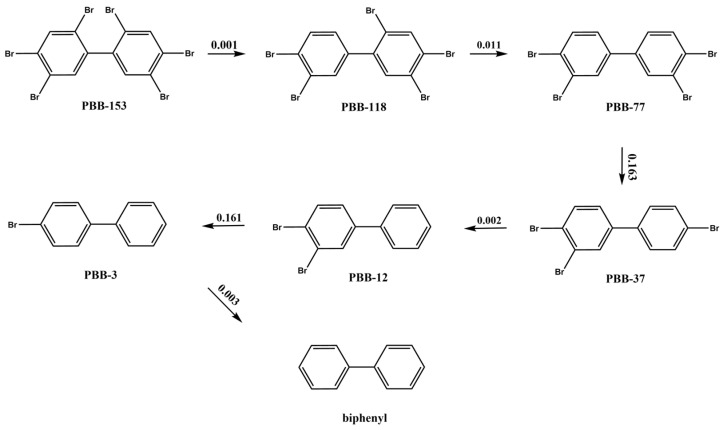
Extrapolation of the photodegradation reaction pathway of PBB-153 based on the minimum energy barrier of the debromination reaction.

Photochemical reactions are important for the degradation of halogenated aromatic compounds, with dehalogenation being the dominant mechanism [15]. In this study, the energy barriers of the debromination pathway at each point of PBB-153 were calculated to deduce the photodegradation pathways of PBB-15, PBB-29, and PBB-209 molecules [15,30]. The positive energy barrier value indicates that the reaction is likely to occur, and the higher the energy barrier value, the higher the energy required for the reaction; and the lower the value, the more likely the reaction would occur [31,32,33]. Therefore, in the photodegradation process of PBB-153, the lower the energy barrier value of debromination at different sites on the benzene ring, the higher the photodegradation efficiency. The photodegradation pathway extrapolation graph of PBB-153 was shown in Figure S1. The most likely photodegradation reaction pathway (Figure 1) was extrapolated according to the minimum energy barrier values listed in Table 1. The numbers marked on the arrows in Figure 1 indicate the energy barrier values of the debromination reaction; the lower the energy barrier value, the easier the reaction is to carry out [34].

First, the ortho-site bromine (Br) atoms on both sides of the benzene ring of PBB-153 were removed in turn to produce PBB-118 and PBB-77 molecules, respectively. Second, the meta-site and para-site bromines on one side of the benzene ring of the PBB-77 molecule were removed in turn to produce PBB-37 and PBB-12 molecules, respectively, with the debromination reaction producing PBB-37 occurring more easily. Finally, the meta-site and ortho-site Br atoms of PBB-12 were successively removed in turn to form PBB-3 and biphenyl (BP).

As shown in Figure 1, the Br atoms of PBB-153 were located at the 2 (ortho-), 4 (para-), and 5 (meta-) sites of the biphenyl ring structure. The order of their debromination reactions was ortho, meta, and para, which differed from the order of the photolytic debromination of PBB-29 (para, ortho, and meta), where Br atoms were distributed on the 2 (ortho-), 4 (para-), and 5 (meta-) sites of the unilateral benzene ring structure [16]. These results suggested that the photolytic debromination order of PBBs changed when both benzene rings are substituted with Br. Experimental and DFT studies showed that the para-bromine substituent in PBB-29 is preferentially removed under UV light. This was attributed to the longer C-Br bond length in the excited state (S1), lower bond dissociation energy (BDE), and higher Mulliken charge on the para-bromine compared to ortho/meta positions, which aligned with the observed para-ortho-meta sequence [15]. For heavier congeners (e.g., hexa- or heptabrominated PBBs), the debromination sequence shifted to ortho-meta-para. This was due to increased steric hindrance and electron-withdrawing effects from multiple bromines, which redistributed molecular charges and destabilized ortho/meta positions more than para [19,35,36]. In highly brominated systems, steric crowding destabilized ortho positions, while electron-withdrawing bromines reduced electron density at meta positions, accelerating their cleavage [37].

#### 2.1.2. Extrapolation of Oxidative Conversion Pathways for the PBBs Combustion

**Figure 2:**
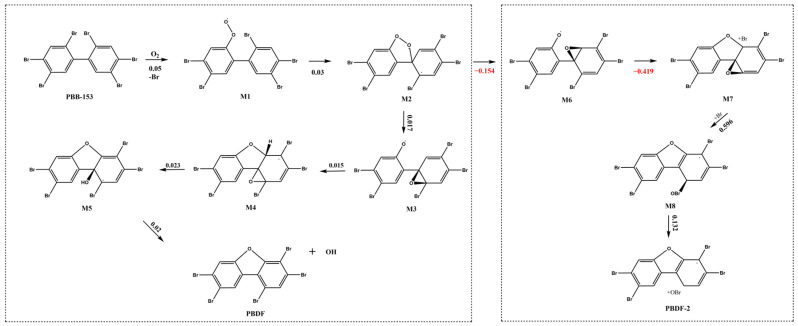
Extrapolation of the reaction path of PBB-153 combustion oxidation.

In this study, the reaction pathway for the combustion oxidation of PBB-153 was deduced based on the PBB-4 oxidation reaction, which forms 4-monobromodibenzofuran and dibenzofuran, as described by Altarawneh et al. (Figure 2) [14]. The combustion oxidation reaction pathway of PBB-4 was obtained from Altarawneh et al. [14]. First, O2 replaced the neighboring Br atom of the single benzene ring of PBB-153 to form a superoxide radical (M1 molecule). Subsequently, an O atom was attached to site 1 of the other benzene ring to form a pentacyclic ring (M2 molecule) under oxidation conditions. M2 molecule underwent O-O bond breaking, pentacyclogenesis, and dihydroxylation to produce the final product, PBDF. The M2 molecule underwent O-O bond cleavage, generating an oxygen radical at the ortho position of one benzene ring, while the other oxygen atom formed a three-membered ring with the brominated site on another benzene ring to yield M3. The oxygen radical in M3 coupled with a hydrogen atom at the ortho position of the neighboring benzene ring to form a pentacyclic ring structure (M4). In M4, the oxygen atom in the three-membered ring then combined with the adjacent hydrogen atom to generate a hydroxyl group, resulting in the formation of M5. Finally, the elimination of the hydroxyl group from M5 produced the PBDF, which was the combustion-derived oxidation product of PBB-153.

Another possible pathway was that the O atom of M2 molecule might also combine with site 6 to form a ternary ring, and the oxygen radical could replace the Br atom at site 2 to form a pentacyclic ring. However, the energy barrier values calculated for this pathway in this study were negative, indicating that this reaction pathway was unlikely to occur. Studies have shown that para-substituted molecules are less stable than ortho-substituted molecules in forming pentacyclic products such as PBDFs [38]. In compounds containing multiple bromine atoms, the electron density around the bromine atoms is higher, indicating that the bromine atoms can attract more electron clouds [39,40]. The difficulty of debromination of PBBs increases with the increase of the number of bromine atoms. This is because highly brominated biphenyl molecules have higher electrophilicity, resulting in a more stable molecular structure [41]. Both benzene rings of PBB-153 molecule are replaced by bromine atoms, so there is an unstable path in the formation of pentacyclic products. The ortho-bromine atoms are more likely to form epoxy structures due to high electron density. In addition, PBB-153 is a highly brominated biphenyl compound, so the process of debromination during combustion oxidation reaction is relatively difficult to occur [42]. In conclusion, the O atom of PBB-153 tended to bind to sites not substituted with Br atoms during the formation of the pentacyclic ring of PBBs, suggesting that higher energies are required for the ring closure reaction if the benzene ring is substituted with multiple Br atoms. When the benzene ring contains multiple bromine substituents, the energy required for ring closure increases significantly due to the decrease of aromaticity [43], electron effect [44], steric hindrance [45], thermodynamic, and kinetic factors [44]. This suggests that combustion oxidation of PBBs with multiple brominated substituents into pentacyclic rings requires higher reaction conditions (such as temperature, pressure, or catalyst) to overcome these energy barriers.

#### 2.1.3. Analysis of PBBs Microbial Reductive Debromination Conversion Pathways

**Figure 3:**
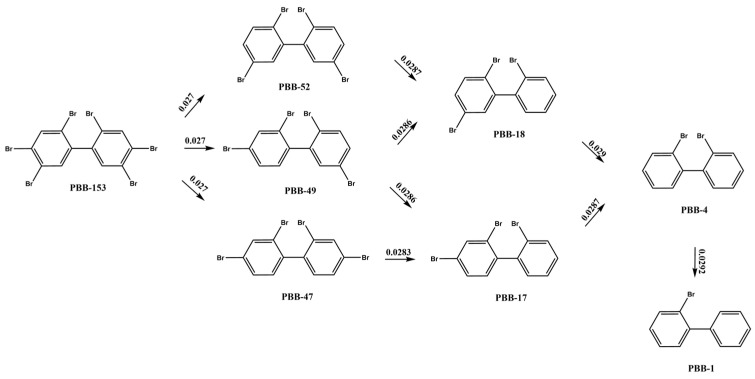
Microbial reductive debromination reaction pathway of PBB-153.

Studies have shown that because of the small steric hindrance, the meta- and para-bromine atoms of PBBs are more easily utilized by microorganisms, and thus preferentially dibrominated [46]. The reductive debromination pathway of PBB-153 by anaerobic microorganisms was deduced, as shown in Figure 3. PBB-153 was reduced to produce three tetrabromobiphenyl congeners (i.e., PBB-47, PBB-49, and PBB-52), which is consistent with the research results of Morris et al. [17]. The common structural factor of PBB-47, PBB-49, and PBB-52 molecules is the presence of ortho-bromine atoms in both benzene rings. They are the products of debromination of the meta- or para-site of PBB-153, as these two sites are more easily utilized by microorganisms. PBB-47 and PBB-49 were debrominated to produce PBB-17, whereas PBB-49 and PBB-52 were debrominated to produce PBB-18. PBB-17 and PBB-18 molecules were both tribromobiphenyl congeners, and the debromination reaction mainly produced PBB-4, a dibromobiphenyl with the ortho-positions of both benzene rings substituted. Finally, PBB-4 underwent single- and double-debromination reactions to sequentially produce PBB-1 and biphenyl. The order of microbial reductive debromination was opposite to that of photoreductive debromination, indicating that microbial reductive action had a certain tendency to select the sites of PBBs, that is, preferentially acting on the meta- and para-positions. Photolysis relies on light energy to cleave C-Br bonds; the energy barrier of photodegradation showed that the sequence of photodegradation and debromination of PBB-153 was ortho-meta-para [15]. Microbial degradation occurs in complex matrices, where molecular orientation and bioavailability influence reactivity. Hydrophobic interactions and enzyme-substrate binding preferences may further bias meta-debromination [47]. Besides, the meta- and para-bromine atoms of PBBs are more easily utilized by microorganisms due to the small steric hindrance [24,46]. Thus, the order of debromination during microbial reduction is the opposite of that observed during photolysis.

#### 2.1.4. Analysis of the PBBs Biometabolic Transformation Pathway

**Figure 4:**
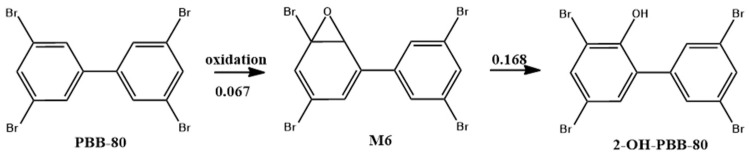
Extrapolation of the biometabolic reaction pathway of mono-hydroxylation of PBB-80.

The most common biometabolic reactions of PBBs were mono- and di-hydroxylation, whereas debromination reactions were less frequent. Congeners with both para-sites substituted with Br atoms were more difficult to metabolize by organisms [30]. PBB-153 had Br atoms on the para-sites of both benzene rings, indicating that it was more difficult to metabolize by organisms. Safe et al. provided a hydroxylation pathway for PBBs metabolism, where epoxidation occurred, and the O-C bond was broken to generate hydroxyl groups [48]. Previous studies have used human liver microsomal enzymes to metabolize PBB-80 in vitro and detected ortho-hydroxyl metabolites without providing the corresponding reaction pathway [49]. This study was based on that of Zhang et al., who inferred the main pathway for PBB-80 monohydroxylation (Figure 4) [49].

As shown in Figure 4, the benzene ring of PBB-80 was first oxidized to generate an epoxide bond, and the ring bond was broken to generate a hydroxyl group. Double-hydroxylated PBBs conversion products could be produced when both benzene rings were oxidized. The energy barrier for PBB-80 to generate the M6 intermediate was lower, making the reaction easier. This indicates that PBB-80 was easily oxidized and metabolized by the organism, while both benzene ring para-sites of PBB-80 were not substituted with Br atoms. This conclusion further confirms that PBBs are more easily oxidized without Br atom substitution at the para sites of the benzene ring.

**Figure 5:**
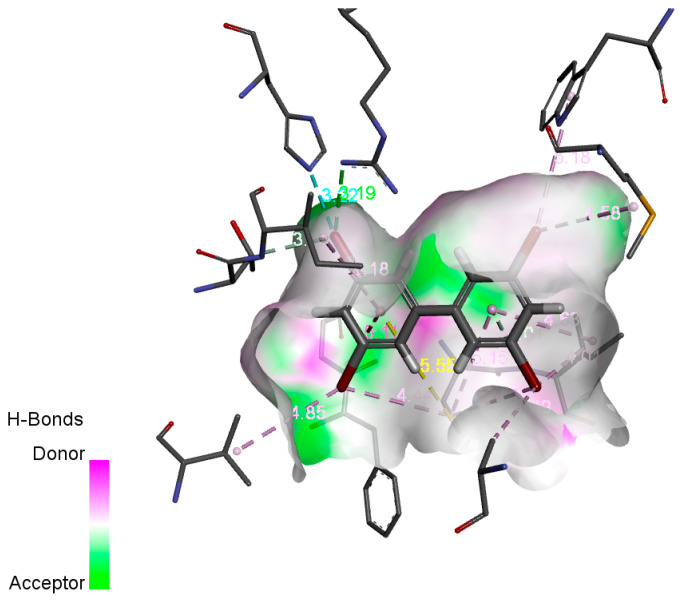
Receptor-ligand non-bonding interactions diagram of PBB-80 biometabolism process.

The potential biometabolic mechanism of PBB-80 was analyzed by molecular docking using the biometabolic protein P450 enzyme as the receptor and PBB-80 as the ligand (Figure 5). The dashed lines in the figure showed the non-bonding interactions between the receptor and ligand. The non-bonding interactions between the P450 enzyme and PBB-80, including hydrogen bonding, hydrophobic interactions, etc., facilitated the enzymatic oxidation of PBB-80, thereby promoting the production of ortho-hydroxylated metabolites. P450 enzyme was more likely to work at sites that were not substituted by Br atoms [50]. In the process of biometabolism, substituents on benzene ring can affect the selectivity of metabolic enzymes [51]. Bromine atom is a strong electron-attracting group, and its introduction can significantly change the electron distribution of the benzene ring [52]. In addition, the introduction of bromine atoms will change the spatial conformation of the benzene ring, and its substitution position will affect the stereochemical properties of the benzene ring. This change in spatial conformation may affect the binding mode of the benzene ring with oxidants or metabolic enzymes [53]. If the para-site is not replaced by bromine, the benzene ring may be more easily recognized by metabolic enzymes, thus speeding up the metabolic process [54]. There were non-bonding forces on the ortho-sites (2, 2′) of the two benzene rings, verifying that PBB-80 was metabolized to generate ortho-site hydroxylation products [49].

#### 2.1.5. Summary of Transformation Products of PBBs and Their Substitutes

The transformation (i.e., photodegradation, combustion oxidation, microbial reduction, and biometabolic pathways) products of PBBs and previously modified environmentally friendly and easily detectable PBB-153 substitutes: 2-C2H5-4-(CH2)2NO2-PBB-153 (D1) [55], 4-COOH-5-NO-PBB-153 (D2) [56], and 5-NO-5′-OCN-PBB-153 (D3) [57] were reviewed and summarized in this study, as shown in Table 2. PBB-1, 2, 3, 9, 15, 30, 77, 103, 153, 154, 169, and 209 have been detected in the e-waste dismantling site [31,58]. PBB-1 might originate from the photodegradation and microbial reductive debromination reactions of PBBs, whereas PBB-2, 3, and PBB-77 may be generated from photodegradation reactions. PBB-3, PBB-30, PBB-1, and PBB-153 were the most frequently detected PBB monomers in the atmosphere, soil, groundwater, and sediments, respectively, at garbage dismantling sites. Thus, photodegradation is speculated to be the most important environmental degradation pathway for PBBs.

In addition, various PBBs have been detected in cancer patients living near e-waste sites, especially those with high concentrations (e.g., PBB-2, 15, 30, 29, 52, 80, and 153). Among these, PBB-2 likely originated from the photodegradation of PBB-29. PBBs detected in cancer patients living near e-waste sites are representative congeners with higher concentrations and greater risks. These molecules may originate from the photodegradation and microbial transformation processes of other congeners, and can undergo further transformations in the environment, posing additional potential risks. Inferring their sources and transformation pathways and evaluating the transformation risks are of great significance for the study and regulation of environmental risks of PBBs. PBBs exhibit high lipophilicity, enabling their rapid permeation through lipid barriers into human tissues [59]. The primary exposure pathways of PBBs include dietary intake, inhalation, and dermal contact [60]. Scientific investigations have revealed that PBBs accumulate preferentially in adipose tissue, followed by organs such as the liver and kidneys [61]. These compounds demonstrate transplacental transfer from maternal circulation to developing fetuses and are subsequently excreted through breast milk to infants [59]. This intergenerational transmission mechanism facilitates the perpetuation of PBBs across generations, thereby enhancing their persistence in human populations. The biomagnification effect of PBBs within food chains results in progressive concentration elevation at higher trophic levels, particularly in humans as apex consumers [62]. Notably, PBBs demonstrate significant bioaccumulative potential, with human elimination half-lives estimated between 13 and 29 years [41,63]. They were not susceptible to debromination by metabolic reactions in living organisms [28]; thus, PBBs detected in humans mainly originated from environmental ingestion by exposure. The immunotoxic and endocrine-disrupting properties of PBBs further contribute to their persistence [64]. Experimental evidence indicates that PBBs disrupt thyroid hormone homeostasis, adversely affecting neurodevelopmental processes and immune functionality [3,4]. Moreover, PBB metabolites may exacerbate metabolic retention through the induction of hepatic enzyme activities [65]. These toxicodynamic interactions potentially amplify the bioaccumulation and long-term persistence of PBBs in human biological systems. PBBs might also be biometabolized to hydroxylated products, which posed more serious hazards to humans [66].

### 2.2. Biotoxicity Evaluation of PBBs and Their Substitutes’ Transformation Products Based on Toxicokinetics

Because PBBs are environmentally persistent and bioconcentrated, they may pose potential toxicity risks to organisms. Therefore, in this study, the biological toxicity risks of PBBs and their substitutes transformation products were evaluated based on their toxicokinetics. The toxicokinetic approach can be used to evaluate the rodent carcinogenicity, rodent toxicity, mutagenicity, potential developmental toxicity, skin and eye irritation, skin sensitization, and aquatic toxicity of contaminants. The probability values of toxicity ranged from 0 to 1 and were divided into three probability intervals: 0–0.3, 0.3–0.7, and 0.7–1.0 [30].

#### 2.2.1. Evaluation of Mutagenicity and Rodent Carcinogenicity of PBBs and Their Substitutes’ Transformation Products

Toxicokinetic evaluations of rodent carcinogenicity were based on the NTP and FDA data sets in Discovery Studio 4.0 software (Table S1). The NTP data set contained four sections for non-carcinogenicity and carcinogenicity in female mice, male mice, female rats, and male rats. The FDA data set included non-carcinogenicity and single/multi-site carcinogenicity in female mice, male mice, female rats, and male rats.

The results of the rodent carcinogenicity evaluation based on the NTP data set showed that all PBB parent molecules were carcinogenic to female mice, except for the PBB substitutes D1 and D3. PBDF was the most carcinogenic compound in female mice, with a probability value of 0.775. Overall, the carcinogenicity of the combustion oxidation products of the parent molecules in female mice tended to increase. The summarized PBBs and their transformation products were carcinogenic to male mice, with probability values ranging from 0.603 to 0.849. These results indicate that the carcinogenic risk to male mice induced by the screened pollutants was significant. Among these compounds, PBB-101 exhibited the most prominent carcinogenic ability in male mice.

The lower brominated congeners (PBB-1, PBB-2, PBB-3, PBB-4, and PBB-15) were not carcinogenic to female rats. However, their debromination products (biphenyl and DF) could induce carcinogenic risk in female rats, with DF having the highest probability of carcinogenic risk (0.657). The remaining parent molecules were carcinogenic to female rats. The probability of carcinogenicity in female rats of all combustion oxidation products tended to increase. No female rats were carcinogenic for metabolized hydroxyl products, while secondary metabolites (i.e., 4′-Br-3-MeO-4-OH-biphenyl and 4-Br-3-MeO-4′-OH-biphenyl) showed increased carcinogenicity. Except for PBB-4-D2, all transformation products of PBBs and their substitutes exhibited carcinogenic effects on male rats, with D3-A1 having the highest carcinogenic risk probability of 0.826. There was a general trend of increased carcinogenicity in male rats across the different transformation products of PBBs and their substitutes.

According to the rodent carcinogenicity evaluation using the FDA data set, D1 and all its transformation products, along with most parent molecules (excluding D3) and their debromination and biometabolites, were not carcinogenic. Both biphenyl and its combustion oxidation products were carcinogenic and affected multiple target sites. In male mice, D2 and its transformation products were not carcinogenic. The debromination products of various parent molecules such as PBB-29, PBB-80, and PBB-153 exhibited carcinogenicity at single or multiple targeting sites. All parent molecules and their transformation products, excluding biphenyl and most of the low-bromination substitutes, were carcinogenic in female rats. The molecule 4-Br-3-MeO-4′-OH-biphenyl had the highest probability (0.623) of affecting multiple sites, while PBB-37 exhibited a higher probability (0.512) of single-site carcinogenicity. In male rats, the trend of the carcinogenicity of PBBs and their substitutes mirrored that observed in female rats. However, some of the metabolites were not carcinogenic. All carcinogenic molecules in male rats targeted a single site, with the combustion product, PBB-15-B1, having the highest carcinogenicity risk.

Except for PBB substitutes, all PBB parent molecules, as well as their debromination and biometabolites, were not mutagenic. However, biphenyl and all the combustion oxidation products of the PBB parent molecules exhibited mutagenic effects. DF posed the highest mutagenic risk, with a probability value of 0.805.

In conclusion, transformation products of PBBs and their substitutes are carcinogenic in rodents. The combustion oxidation products present elevated carcinogenic and mutagenic risks. This suggests that when incineration is used to treat e-waste containing PBBs, the carcinogenic and mutagenic risks of the resultant products warrant careful consideration.

#### 2.2.2. Biotoxicity Evaluation of PBBs and Their Substitutes’ Transformation Products

In this study, the biological toxicities of PBBs and their substitutes’ transformation products on rats, fathead minnow, and *Daphnia magna* were assessed. The evaluation parameters included oral LD_50_, the maximum tolerated dose in rats administered through feed/water and gavage, LC_50_ in rats upon inhalation, chronic oral lowest observed adverse effect level in rats, LC_50_ values for fathead minnow, and EC_50_ values for *Daphnia magna*. The detailed results are presented in Table S2. According to toxicity classification, LD_50_ values were categorized: ≤ 0.005 g/kg as extremely toxic (++++), 0.005~0.05 g/kg as highly toxic (+++), 0.05~0.3 g/kg as moderately toxic (++), 0.3~2 g/kg as slightly toxic (+), and >2 g/kg as practically non-toxic (−).

Oral administration, maximum tolerated dose (feed/water), and maximum tolerated dose (gavage) were selected as acute toxicity evaluation parameters in rats. The toxicity classifications of the PBBs’ parent molecules and their transformation products showed increasing severity, ranging from low/non-toxic to toxic/high/very toxic. Generally, the debromination products of the PBBs’ parent molecules exhibited decreasing toxicity, whereas the biological metabolites and combustion oxidation products showed increasing toxicity.

Acute toxicity evaluations indicated higher biological toxicity of the three substitutes of PBB-153 compared to other PBB molecules. Both the maximum tolerated dose of PBB-153 and the remaining parent molecules were highly toxic. Additionally, the maximum tolerated dose (gavage) toxicity of the D2 and D3 molecules and their transformation products significantly increased, all of which were classified as highly toxic. This suggests a higher acute toxicity risk for these substitutes in rats, whereas D1 showed a decreasing trend in the acute toxicity risk.

The chronic toxic effects of the molecules were evaluated for long-term oral toxicity in rats, with PBB-4 and PBB-29 classified as moderately toxic and all other PBB molecules classified as highly toxic. Some debrominated and biometabolites were reduced to toxic, such as biphenyl, PBB-14, PBB-12, 4′-OH-PBB-3, and 4-OH-PBB-3. The combustion oxidation products of PBBs showed an increasing trend in toxicity; for instance, products after combustion of PBB-4 and PBB-29 increased from toxic to highly toxic, indicating a significant biological toxicity that warrants attention, consistent with the high mutagenic and carcinogenic risks associated with the combustion oxidation products of PBBs. The toxicity classification of the PBB-153 substitutes after combustion remained unchanged and was generally consistent with the virtual modification effect of PBB-153 on environmental friendliness.

LC_50_ values for inhalation by rats were graded as extremely toxic (≤2000 mg/m^3^/h), highly toxic (2000–8000 mg/m^3^/h), toxic (8000–40,000 mg/m^3^/h), and low toxic (40,000–80,000 mg/m^3^/h) [67]. The evaluation results showed that most PBBs biometabolites, PBB-153, some of its debromination products, and all transformation products of D2 and D3 molecules (except D3-A3) had highly toxic effects via inhalation, indicating a significant environmental risk through this exposure route. Unlike other toxic effects, the combustion oxidation products of the PBBs molecules showed a decreasing trend in toxicity levels.

The toxicity of PBBs and their substitutes’ transformation products to fathead minnow and *Daphnia magna* was evaluated based on ecotoxicological hazard classification. The hazard rating of LC_50_ and EC_50_ values is as follows: less than or equal to 1 mg/L indicates very high toxicity, 1–10 mg/L indicates high toxicity, 10–100 mg/L indicates medium toxicity, and greater than 100 mg/L indicates low toxicity [68].

The photodegradation and biometabolism products of the biphenyl molecules and some PBB substitutes were less toxic to *Daphnia magna* than the parent molecules. In contrast, the combustion products of specific molecules (e.g., PBB-3, PBB-15, and PBB-80) exhibited elevated toxicity. Overall, the toxicity to *Daphnia magna* decreased for all transformation products compared to the parent molecules. However, the potential ecological risks to aquatic environments cannot be disregarded owing to their high hazard ratings.

#### 2.2.3. Evaluation of Biological Tissue Toxicity and Potential Developmental Toxicity of PBBs and Their Substitutes’ Transformation Products

The toxicokinetic calculations for the biotoxicity and potential developmental toxicity of PBBs and their substitutes’ transformation products are detailed in Table S3. PBB-3, PBB-4, and PBB-29 exhibited no potential biotoxicity, whereas the remaining PBBs exhibited potential biotoxicity. Most PBB debromination products did not exhibit potential developmental toxicity, whereas combustion oxidation products and biometabolites demonstrated higher potential developmental toxicity. Notably, the dihydroxy metabolite 2,2′-OH-PBB-80 of the PBB-80 molecule presented the highest potential developmental toxicity risk (toxicity probability 0.713), which was 18% higher than that of the parent molecule. Furthermore, developmental toxicity increased in the biometabolites of all PBBs and most combustion oxidation products, except for PBB-80 and D1 molecules, indicating potential risks from the combustion oxidation and biometabolism processes of PBBs.

Skin risks include irritation and sensitization, classified as mild/moderate and strong/weak, respectively. As shown in Table S3, most PBBs (including their debromination and combustion oxidation products) exhibited a high probability of skin irritation (all > 0.95). PBDF, the PBB-153 combustion oxidation product, showed the strongest irritation (probability 0.995), classifying it as mild irritation. Biometabolites cannot cause skin irritation. Except for D1 and its combustion oxidation product, none of the PBB-153 substitutes or transformation products irritated the skin, demonstrating that virtual molecular modification effectively mitigates the skin-irritation effects of PBBs. PBBs not only irritate the skin but also sensitize it. The combustion oxidation products of PBB-3, PBB-4, PBB-29, D1, and D2 were non-sensitizing. The remaining transformation products and all PBBs exhibited high probabilities of skin sensitization (0.749–0.883), all strongly sensitizing. Overall, PBBs transformation products showed a higher probability of strong skin sensitization than the parent molecules, whereas biometabolites showed some sensitization potential.

Eye irritation was classified as mild/moderate-severe and moderate/severe levels. Biphenyl and D3-C2 caused no eye irritation, whereas other molecules exhibited eye irritation probabilities ranging from 0.975 to 1. Most PBBs biometabolites showed moderate-severe eye irritation levels. Specifically, 4-OH-PBB-13, PBB-29-D1, and PBB-29-D2 were multi-site irritants, indicating a higher risk of eye irritation caused by biometabolites. The PBB-153-D1 molecule and its substitutes caused only mild eye irritation, suggesting that virtual molecular modifications could mitigate the potential eye irritation risks from PBBs.

In summary, PBBs and their substitution products pose high toxicity risks to organisms, including carcinogenicity, mutagenicity, developmental toxicity, and other effects. They present a high environmental risk to aquatic organisms such as fish and *Daphnia Magna*, primarily due to the combustion and oxidation products of PBBs. The assessment of health risks from exposure to PBBs and their transformation products is crucial, considering their multiple toxic effects on humans.

### 2.3. Potential Human Toxicity Risk Assessment of PBBs and Their Substitutes Based on 3D-QSAR Models

#### 2.3.1. Construction and Evaluation of 3D-QSAR Models of Human Toxicity of PBBs and Their Substitutes

PBBs pose multiple toxicity risks in humans, including carcinogenicity, developmental toxicity, hepatotoxicity, epigenotoxicity, neurotoxicity, and immunotoxicity [5,9,69,70,71,72]. 3D-QSAR models were constructed to assess these risks, and the model parameters are listed in Table 3. The q^2^ values for the constructed 3D-QSAR models were 0.827, 0.818, 0.836, 0.895, 0.807, and 0.825 (exceeding 0.5), indicating robust predictive abilities [73]. The R^2^ values were 0.994, 0.966, 0.990, 0.991, 0.995, and 0.993, all exceeding 0.9, demonstrating excellent fitting capabilities [74]. The r^2^_pred_ values for the constructed 3D-QSAR models were 0.853, 0.856, 0.784, 0.633, 0.915, and 0.810. All r^2^_pred_ values exceeded 0.6, indicating strong external predictive abilities [58]. Compared to other published 3D-QSAR studies, the current models demonstrate superior predictive capabilities. Specifically, when examining the relationship between the chemical structure of flavonoid analogs and their ability to inhibit xanthine oxidase [75], developing safer and more potent alpha-glucosidase inhibitors [76], and studying mutant isocitrate dehydrogenase 1 inhibitors [77], previous studies reported q² values of 0.77, 0.60, and 0.765, respectively, along with R² values of 0.98, 0.958, and 0.980. In contrast, our models exhibit higher q² and R² values. For external validation results, the r^2^_pred_ values of the 3D-QSAR models of sulfonamide HBV core protein allosteric modulators [78], novel Bromodomain-containing protein 4 inhibitors [79], and selective antagonists of the N-Methyl-D-Aspartate receptor subunit 2B [80] were 0.628, 0.629, and 0.613, respectively. Our models exhibit higher r^2^_pred_ values, indicating enhanced performance. In general, the established 3D-QSAR models in this study exhibited excellent predictive consistency and discriminative capacity, providing reliable toxicological risk predictions for both parent PBBs and their transformation products in human exposure scenarios.

**Figure 6:**
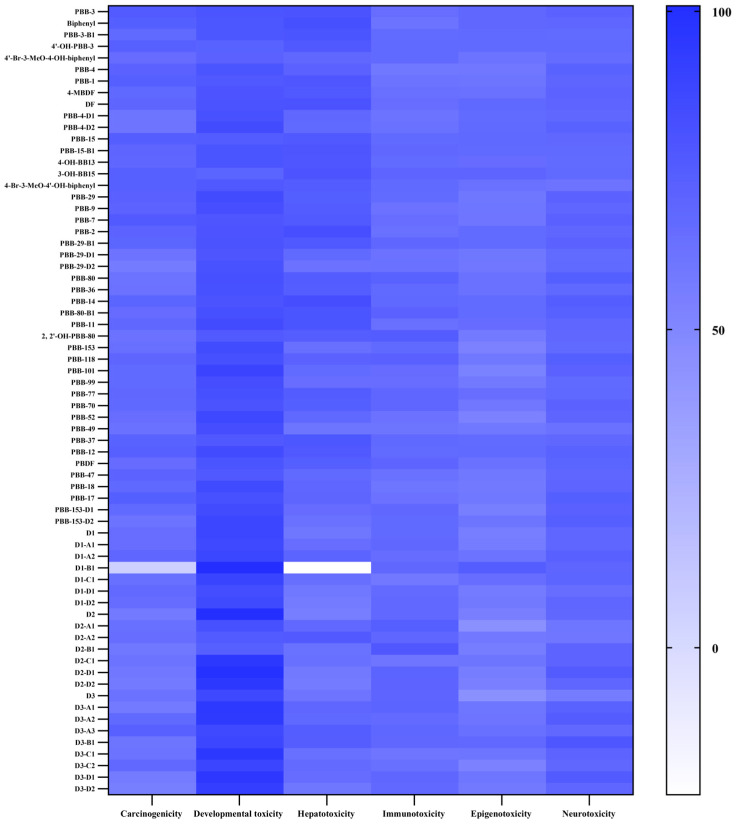
Evaluation of the potential human risk of PBBs and their substitutes.

In summary, the constructed CoMFA models for different human toxicity risks of PBBs met the validation criteria; thus, the models could be used to predict the human toxicity risks of PBBs and their substitute transformation products. The six 3D-QSAR models enabled the prediction and assessment of diverse human toxicity risks associated with PBBs and their substitutes’ molecular transformation products using Risk Characterization Values (Figure 6), where higher values indicate more significant toxicity risks.

#### 2.3.2. Potential Carcinogenic Risks of PBBs and Their Substitutes’ Transformation Products

The human carcinogenic potential of PBBs exposure, focusing on thyroid cancer, was analyzed in this study. The risk characterization values for the human carcinogenicity of PBBs and their alternative environmental transformation products ranged from 6.47 to 76.50. PBB-7 showed the highest carcinogenic risk, whereas D1-B1 showed the lowest. Environmental debromination products of PBB-153 showed an increase in carcinogenic risk from −1.96% to 17.91%, compared with PBB-153. Notably, lower brominated biphenyls exhibited relatively higher human carcinogenic risk characterization values, indicating an increase in human carcinogenic risk with the environmental photodegradation transformation of PBBs. Furthermore, PBB-80, PBB-153, and PBB-153-D2 showed an increasing trend in carcinogenic risk, whereas the others showed a decreasing trend. Overall, PBB-4 and its transformation products demonstrated higher carcinogenic risk values. The overall human carcinogenic risk of PBBs substitutes and their transformation products was lower than that of PBBs parent molecules, consistent with the objective of the virtual modification of PBBs.

#### 2.3.3. Potential Developmental Toxicity Risks of PBBs and Their Substitutes’ Transformation Products

The characterization values for the human developmental toxicity risk of PBBs and their substitutes’ transformation products were higher than those for other toxicity risk effects. PBB-153 and its substitutes exhibited the highest potential human developmental toxicity risk among the parent molecules, with characterization values ranging from 70.53 to 100.87. In contrast, PBB-15 showed the lowest risk of human developmental toxicity. Generally, the human developmental toxicity of PBBs increased with the number of Br atom substitutions, but the transformation products of the PBBs parent molecules showed a decreasing trend in human developmental toxicity. For instance, the transformation products of PBB-3, PBB-29, and D2 exhibited decreased human developmental toxicity risk. Taking PBB-153 as an example of high developmental toxicity risk, its debromination products PBB-101, PBB-52, and PBB-18, and the biometabolite PBB-153-D2, showed elevated developmental toxicity risk, with PBB-101 exhibiting the highest toxicity among them.

#### 2.3.4. Potential Hepatotoxicity Risks of PBBs and Their Substitutes’ Transformation Products

Human hepatotoxicity characterization values for transformation products of PBBs and their substitutes ranged from −23.02 to 82.786. D1-B1 and PBB-14 exhibited the lowest and highest potential for human hepatotoxicity, respectively. Highly brominated PBBs showed high hepatotoxicity, whereas PBB-153, its substitutes, and their transformation products showed relatively low hepatotoxicity, in contrast to the trend observed for human developmental toxicity. The hepatotoxicity of the PBB-153 substitutes and their transformation products was significantly lower than that of PBB-153 and its transformation products, indicating that virtual molecular modifications can serve as a theoretical tool for new material development. Notably, the combustion oxidation product D1-B1 of D1 exhibited a negative value for human hepatotoxicity characterization, suggesting low binding affinity to relevant proteins characterizing human hepatotoxicity and minimal toxic effects.

#### 2.3.5. Potential Epigenotoxicity Risks of PBBs and Their Substitutes’ Transformation Products

Characterization values for the potential human epigenotoxicity of PBBs and their substitutes’ transformation products ranged from 44.31 to 74.73. Most transformation products exhibited increased human epigenotoxicity risk compared with the parent molecules. This suggests that there is concern regarding the potential for epigenetic toxicity in humans from exposure to the transformation products of PBBs in the environment. Except for the D1-B1, transformation products of PBB-153 and its substitutes showed lower epigenotoxicity than the other molecules, suggesting that virtual molecular modifications can mitigate potential human epigenotoxicity risks from contaminants.

#### 2.3.6. Potential Human Neurotoxic Risks of PBBs and Their Substitutes’ Transformation Products

Potential human neurotoxicity characterization values of PBBs and their substitutes’ transformation products ranged from 56.54 to 78.24. D3 and its combustion oxidation product, D3-B1, exhibited the lowest and highest toxicity risks, respectively. Except for PBB-3, PBB-4, PBB-15, and PBB-80, the combustion oxidation products of all PBBs showed an increasing trend in terms of human neurotoxicity risk. Overall, the human neurotoxicity of most PBBs transformation products decreased, with the photodegradation products D2-A1 and D2-A2 of D2 molecule showing decreases of 12.5% and 12.82% in human neurotoxicity, respectively.

#### 2.3.7. Potential Immunotoxicity Risks of PBBs and Their Substitutes’ Transformation Products

Potential human immunotoxicity characterization values for PBBs and their substitutes’ transformation products ranged from 58.67 to 77.35. The D1-C1 microbial reduction product of D1 exhibited the lowest toxicity, whereas the combustion oxidation product D2-B1 of D2 exhibited the highest toxicity. Overall, both the combustion oxidation products and biometabolites of PBBs showed an increasing trend of human immunotoxicity.

## 3. Materials and Methods

### 3.1. Calculation of Energy Barriers for PBBs Transformation Pathways—Gaussian Calculation

The energy barriers for the degradation pathways (i.e., photodegradation and combustion oxidation) of the most commonly used PBB (i.e., PBB-153) were calculated using Gaussian 09 software at the level of b3pw91/6–31G based on density functional theory (DFT) [14,15,16]. The bond length of the transition-state molecule was extended by up to one-third compared to that of the original molecule. Positive energy barrier values indicated that the reaction could occur, and the smaller the value, the more likely the reaction would occur [31]. The energy barrier values could be used to determine whether the intermediate products could be easily produced during the reaction process, thus inferring whether the pathway transformation products PBBs and their substitutes could be easily generated.

### 3.2. Animal Toxicity Evaluation of PBBs and Their Transformation Products—Toxicokinetic

Discovery Studio 4.0 software includes a TOPKAT prediction module that can be used to evaluate the biological toxicity risks of molecules. The biological toxicity risks include rodent carcinogenicity (NTP and FDA data sets), mutagenicity (Ames test), rat chronic oral lowest observed adverse effect level, rat oral LD_50_, maximum tolerated dosage, inhalational LC_50_, developmental toxicity potential, skin sensitization, skin irritancy, ocular irritancy, fathead minnow LC_50_, and *Daphnia magna* EC_50_ [81].

### 3.3. Prediction of PBBs and Their Transformation Products’ Human Toxicities—Molecular Docking and 3D-QSAR Method

PBB molecules were docked with proteins using Discovery Studio 4.0 software. The LibDock score of the docked complex represents different toxic risks to the human body (e.g., carcinogenicity, reproductive developmental toxicity, hepatotoxicity, epigenetic toxicity, neurotoxicity, and immunotoxicity) under the LibDock module. “Docking Preferences” were set to “User Specified”, the “Max Hits to Save” was set to 1, and then the docking scores were obtained. The higher the docking score, the stronger the ability of the molecule to bind to the protein, indicating a greater risk of toxicity to humans [82]. The protein structures used for molecular docking were obtained from the PDB database (RCSB PDB: Homepage). The PDB IDs representing human carcinogenicity, reproductive developmental toxicity, hepatotoxicity, epigenotoxicity, neurotoxicity, and immunotoxicity were 2riw, 2yja, 5v0l, 4wxx, 6u3p, and 4grl, respectively.

Human carcinogenicity, reproductive developmental toxicity, hepatotoxicity, epigenotoxicity, neurotoxicity, and immunotoxicity characterization values of PBBs were used as dependent variables, and the molecular structures of PBBs were used as independent variables to construct six 3D-QSAR models. The molecular structure of PBBs was drawn and minimized using Sybyl-x2.0 (Tripos, St. Louis, Mo, USA). The charge carried by the molecules was the Gasterger–Hückle charge. The Tripos molecular force field was used with an energy convergence criterion of 0.005 kcal/mol, and 10,000 iterations were performed to obtain the most stable molecular conformation. The common skeletons of the optimized molecules were superimposed, and PBB-153 was selected as the template molecule because of its high application and environmental detection frequency.

Twenty-four PBBs were randomly selected as the training and the test sets in a ratio of 3:1, and six 3D-QSAR models of PBBs were established using Sybyl-x2.0 software and analyzed using the partial least squares method [83]. The Leave-One-Out method was used for cross-validation of the training set compounds, and the cross-validation coefficient q^2^ and the best principal component fraction n were obtained [84]. Regression analysis was used to calculate the non-cross-validation coefficient R^2^, standard deviation SEE, and test value F [85]. The robustness of the models was tested, and their external predictive ability of the model was evaluated, with the evaluation parameters SEP and r^2^_pred_ calculated [86].

## 4. Conclusions

This study inferred and summarized almost 70 transformation products derived from photodegradation, microbial degradation, combustion oxidation, and metabolism of PBBs and their substitutes. The analysis results indicated that these transformation products of PBBs and their substitutes posed high toxicological risks to organisms, exhibiting potential carcinogenic, mutagenic, and developmental toxicity effects. These compounds particularly posed high potential toxicological risks to aquatic organisms, such as fish and *Daphnia magna*, with the primary ecological risk stemming from the combustion oxidation products of PBBs. Among the potential toxicological risks to humans, the developmental toxicity of PBBs and their surrogate transformation products exhibited higher characterization values. Despite the environmental friendliness and functionality of the previously designed PBB substitutes, their parent molecules and byproducts presented potential risks to animals and humans. In addition, the developmental toxicity of PBBs and substitutes’ transformation products seemed to generate more severe risks. Therefore, a more comprehensive risk evaluation should be conducted when designing new flame retardants. This study provides theoretical approaches for studying the toxicity of pollutants represented by PBBs.

## Figures and Tables

**Table 1:** The energy barriers of the PBB-153 molecular photodebromination pathway.

Number of Debromination	Debromination Sites	Energy Barrier/a.u.
1	2	0.001
	4	0.027
	5	0.027
2	2, 4	0.164
	2, 5	0.163
	2, 2′	0.011
	2, 4′	0.164
	2, 5′	0.164
3	2, 2′, 3	0.163
	2, 2′, 4	0.164
4	2, 2′, 3, 3	0.162
	2, 2′, 3, 4	0.164
	2, 2′, 3, 4′	0.002
5	2, 2′, 3, 4′, 3	0.161
	2, 2′, 3, 4′, 4	0.164
6	2, 2′, 3, 4′, 3, 4	0.003

**Table 2:** Summary of transformation products of PBBs and their substitutes.

PBBs	Transformation Products	Transformation Pathways
PBB-3	Biphenyl	Photodegradation
PBB-4	PBB-1, Biphenyl	
PBB-15	PBB-3, Biphenyl	
PBB-29	PBB-9, PBB-7, PBB-3, PBB-2, PBB-1, biphenyl	
PBB-80	PBB-36, PBB-14, PBB-2, Biphenyl	
PBB-153	PBB-118, PBB-101, PBB-99, PBB-77, PBB-70, PBB-52, PBB-49, PBB-37, PBB-12, PBB-3, biphenyl	
D1	D1-A1, D1-A2	
D2	D2-A1, D2-A2	
D3	D3-A1, D3-A2, D3-A3	
PBB-3	PBB-3-B1	Combustion oxidation
PBB-4	4-MBDF, DF	
PBB-15	PBB-15-B1	
PBB-29	PBB-29-B1	
PBB-80	PBB-80-B1	
PBB-153	PBDF	
D1	D1-B1	
D2	D2-B1	
D3	D3-B1	
PBB-3	Biphenyl	Microbial reduction
PBB-4	PBB-1, Biphenyl	
PBB-15	PBB-3, Biphenyl	
PBB-29	PBB-7, PBB-9, PBB-1, Biphenyl	
PBB-80	PBB-36, PBB-14, PBB-11, PBB-2, Biphenyl	
PBB-153	PBB-52, PBB-49, PBB-47, PBB-18, PBB-17, PBB-4, PBB-1	
D1	D1-C1	
D2	D2-C1	
D3	D3-C1, D3-C2	
PBB-3	4′-OH-PBB-3, 4′-Br-3-MeO-4-OH-biphenyl,	Biological metabolism
PBB-4	PBB-4-D1, PBB-4-D2	
PBB-15	4-OH-PBB-13, 3-OH-PBB-15, 4-Br-3-MeO-4′-OH-biphenyl	
PBB-29	PBB-29-D1, PBB-29-D2	
PBB-80	2, 2′-OH-PBB-80	
PBB-153	PBB-153-D1, PBB-153-D2	
D1	D1-D1, D1-D2	
D2	D2-D1, D2-D2	
D3	D3-D1, D3-D2	

**Table 3:** Evaluation parameters of the six constructed 3D-QSAR models.

3D-QSAR Models	q^2^	n	R^2^	SEE	F	SEP	r^2^_pred_
Carcinogenicity model	0.827	10	0.994	0.532	111.695	3.279	0.853
Developmental toxicity model	0.818	5	0.966	0.879	68.755	6.225	0.856
Hepatotoxicity model	0.836	5	0.99	0.94	227.902	4.427	0.784
Epigenotoxicity model	0.895	7	0.991	0.598	158.151	4.318	0.633
Neurotoxicity model	0.807	8	0.995	0.494	220.73	0.868	0.915
Immunotoxicity model	0.825	8	0.993	0.403	156.833	2.276	0.81

## Data Availability

All relevant data of this study are presented. Additional data will be provided upon request.

## Supplementary Materials

The following supporting information can be downloaded at: https://www.mdpi.com/article/10.3390/ijms26041753/s1.

## Abbreviations

The following abbreviations are used in this manuscript:

PBBs	Polybrominated biphenyls
Br	Bromine
PBDF	Polybrominated dibenzofuran
BP	Biphenyl
TOPKAT	Toxicokinetics
