# Supplementary material for: Extrapolation of PBBs Environmental Transformation Mechanisms and Toxicity Risks of Byproducts
Source: Int J Mol Sci. 2025 Feb 19;26(4):1753. doi: 10.3390/ijms26041753 (PMC11855703; doi:10.3390/ijms26041753)
Supplement: Supplementary file 1 [file ijms-26-01753-s001.zip › ijms-3474896-supplementary.pdf]

Supplementary Materials

Extrapolation of PBBs Environmental Transformation Mechanisms and Toxicity Risks of Byproducts

Bohan Xu [1†], Qian Liu [2†], Weihan Cui [1], Li Tao [1], Yuanquan Chi [1], Luze Yang [1,*]

[1] College of Resources and Environment, Jilin Agricultural University, Changchun, 130118, China; xbh2275850139@163.com; 19274302086@163.com; q3364367547@163.com; sourcechi6@163.com

[2] National Engineering Laboratory for Lake Pollution Control and Ecological Restoration, State Environmental Protection Key Laboratory for Lake Pollution Control, Chinese Research Academy of Environmental Sciences, Beijing 100012, China; liuqiankaoyan@163.com

* Correspondence: Yanglz2022@outlook.com, yanglz19@mails.jlu.edu.cn.

† These authors have contributed equally to the study and they receive equal credit.

This SI file contains: 16 Pages; 1 Figure; 3 Tables.

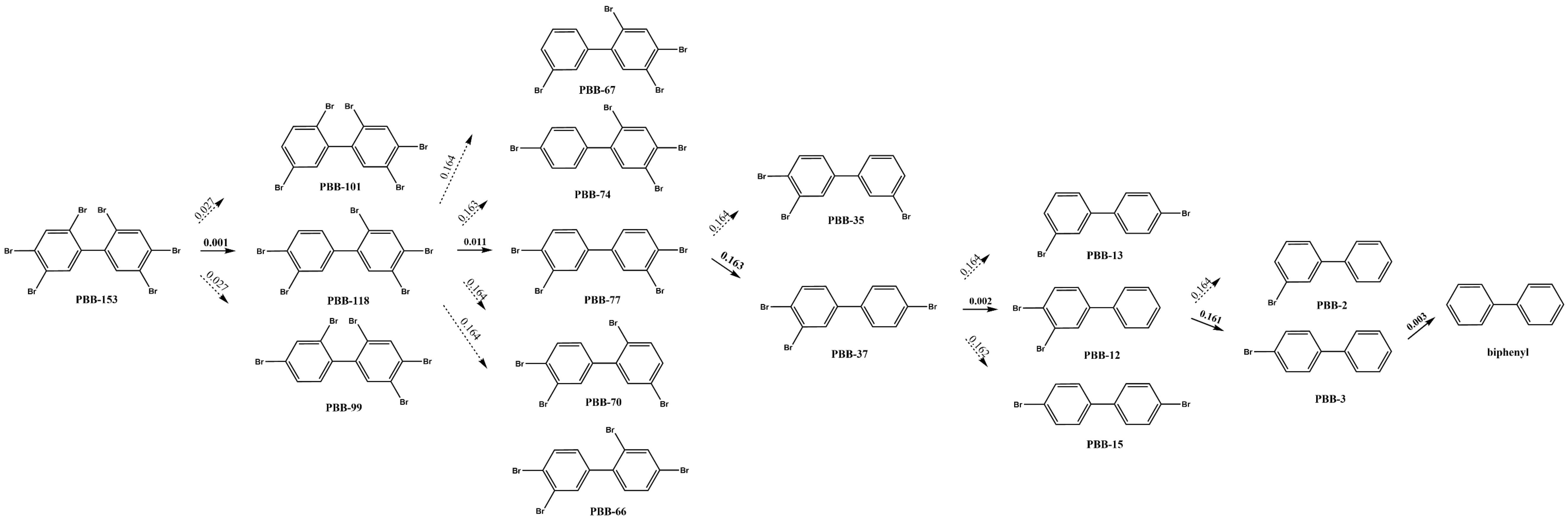

Figure S1. The photodegradation pathway extrapolation graph of PBB-153

Table S1 Evaluation of potential mutagenicity and rodent carcinogenicity o PBBs and their substitutes' f transformation products

	NTP dataset				FDA Data Set								
	Non/carcinogenic in female mice	Non/carcinogenic in male mice	Non-/carcinogenic in female rats	Non-/carcinogenic in male rats	Non/carcinogenic in female mice	Single/multi-site carcinogenicity in female mice	Non/carcinogenic in male mice	Single/multi-site carcinogenicity in male mice	Non-/carcinogenic in female rats	Single/multi-site carcinogenicity in female rats	Non-/carcinogenic in male rats	Single/multi-site carcinogenicity in male rats	Mutagenicity
PBB-3	0.695/carcinogenic	0.713/carcinogenic	0.486/non	0.719/carcinogenic	0.227/non		0.255/non		0.262/non		0.327/non		0.649/non
Biphenyl	0.607/carcinogenic	0.603/carcinogenic	0.540/carcinogenic	0.655/carcinogenic	0.270/carcinogenic	0.441/multi	0.315/carcinogenic	0.192/multi	0.244/non		0.325/non		0.767/mutagenic
PBB-3-B1	0.706/carcinogenic	0.818/carcinogenic	0.609/carcinogenic	0.687/carcinogenic	0.264/carcinogenic	0.405/multi	0.326/carcinogenic	0.147/single	0.449/carcinogenic	0.443/single	0.543/carcinogenic	0.536/single	0.778/mutagenic
4'-OH-PBB-3	0.565/non	0.622/carcinogenic	0.499/non	0.675/carcinogenic	0.259/carcinogenic	0.471/multi	0.240/non		0.368/carcinogenic	0.580/multi	0.359/carcinogenic	0.561/single	0.605/non
4'-Br-3-MeO-4-OH-biphenyl	0.572/non	0.742/carcinogenic	0.536/carcinogenic	0.669/carcinogenic	0.205/non		0.195/non		0.262/non		0.299/non		0.607/non
PBB-4	0.653/carcinogenic	0.734/carcinogenic	0.490/non	0.703/carcinogenic	0.235/non		0.260/non		0.328/carcinogenic	0.520/multi	0.360/carcinogenic	0.517/single	0.669/non
PBB-1	0.754/carcinogenic	0.713/carcinogenic	0.511/non	0.708/carcinogenic	0.220/non		0.228/non		0.286/carcinogenic	0.522/multi	0.327/non		0.633/non
Biphenyl	0.607/carcinogenic	0.603/carcinogenic	0.540/carcinogenic	0.655/carcinogenic	0.270/carcinogenic	0.441/multi	0.315/carcinogenic	0.192/multi	0.244/non		0.325/non		0.767/mutagenic
4-MBDF	0.654/carcinogenic	0.799/carcinogenic	0.625/carcinogenic	0.681/carcinogenic	0.251/carcinogenic	0.348/multi	0.357/carcinogenic	0.149/single	0.438/carcinogenic	0.443/single	0.543/carcinogenic	0.536/single	0.785/mutagenic
DF	0.619/carcinogenic	0.666/carcinogenic	0.657/carcinogenic	0.638/carcinogenic	0.288/carcinogenic	0.404/multi	0.396/carcinogenic	0.164/single	0.388/carcinogenic	0.448/single	0.532/carcinogenic	0.546/single	0.805/mutagenic

PBB-4-D1	0.388/non	0.644/carcinogenic	0.464/non	0.630/carcinogenic	0.206/non		0.207/non		0.270/carcinogenic	0.533/multi	0.326/non		0.637/non
PBB-4-D2	0.340/non	0.611/carcinogenic	0.472/non	0.589/non	0.209/non		0.215/non		0.279/carcinogenic	0.533/multi	0.349/non		0.653/non
PBB-15	0.621/carcinogenic	0.726/carcinogenic	0.494/non	0.701/carcinogenic	0.233/non		0.252/non		0.321/carcinogenic	0.524/multi	0.376/carcinogenic	0.516/single	0.627/non
PBB-3	0.695/carcinogenic	0.713/carcinogenic	0.486/non	0.719/carcinogenic	0.227/non		0.255/non		0.262/non		0.327/non		0.649/non
Biphenyl	0.607/carcinogenic	0.603/carcinogenic	0.540/carcinogenic	0.655/carcinogenic	0.270/carcinogenic	0.441/multi	0.315/carcinogenic	0.192/multi	0.244/non		0.325/non		0.767/mutagenic
PBB-15-B1	0.708/carcinogenic	0.776/carcinogenic	0.537/carcinogenic	0.668/carcinogenic	0.272/carcinogenic	0.407/multi	0.275/carcinogenic	0.158/single	0.368/carcinogenic	0.467/single	0.455/carcinogenic	0.544/single	0.743/mutagenic
4-OH-PBB-13	0.544/non	0.704/carcinogenic	0.438/non	0.710/carcinogenic	0.219/non		0.193/non		0.285/carcinogenic	0.535/multi	0.333/non		0.579/non
3-OH-PBB-15	0.576/non	0.697/carcinogenic	0.426/non	0.728/carcinogenic	0.221/non		0.199/non		0.285/carcinogenic	0.533/multi	0.344/non		0.607/non
4-Br-3-MeO-4'-OH-biphenyl	0.567/non	0.612/carcinogenic	0.561/carcinogenic	0.747/carcinogenic	0.225/non		0.246/non		0.325/carcinogenic	0.623/multi	0.349/non		0.687/non
PBB-29	0.728/carcinogenic	0.766/carcinogenic	0.555/carcinogenic	0.707/carcinogenic	0.226/non		0.249/non		0.281/carcinogenic	0.510/single	0.331/non		0.623/non
PBB-9	0.729/carcinogenic	0.750/carcinogenic	0.509/non	0.695/carcinogenic	0.227/non		0.250/non		0.281/carcinogenic	0.523/multi	0.334/non		0.635/non
PBB-7	0.710/carcinogenic	0.737/carcinogenic	0.493/non	0.711/carcinogenic	0.217/non		0.195/non		0.287/carcinogenic	0.523/multi	0.334/non		0.560/non
PBB-3	0.695/carcinogenic	0.713/carcinogenic	0.486/non	0.719/carcinogenic	0.227/non		0.255/non		0.262/non		0.327/non		0.649/non

PBB-2	0.600/carcinogenic	0.736/carcinogenic	0.505/non	0.696/carcinogenic	0.228/non		0.268/carcinogenic	0.162/single	0.258/non		0.327/non		0.659/non
PBB-1	0.754/carcinogenic	0.713/carcinogenic	0.511/non	0.708/carcinogenic	0.220/non		0.228/non		0.286/carcinogenic	0.522/multi	0.327/non		0.633/non
Biphenyl	0.607/carcinogenic	0.603/carcinogenic	0.540/carcinogenic	0.655/carcinogenic	0.270/carcinogenic	0.441/multi	0.315/carcinogenic	0.192/multi	0.244/non		0.325/non		0.767/mutagenic
PBB-29-B1	0.704/carcinogenic	0.811/carcinogenic	0.629/carcinogenic	0.678/carcinogenic	0.251/carcinogenic	0.369/multi	0.318/carcinogenic	0.153/single	0.400/carcinogenic	0.454/single	0.523/carcinogenic	0.514/single	0.786/mutagenic
PBB-29-D1	0.533/non	0.705/carcinogenic	0.522/non	0.704/carcinogenic	0.218/non		0.242/non		0.313/carcinogenic	0.534/multi	0.351/carcinogenic	0.529/single	0.603/non
PBB-29-D2	0.553/non	0.612/carcinogenic	0.496/non	0.767/carcinogenic	0.213/non		0.248/non		0.272/carcinogenic	0.563/multi	0.333/non		0.592/non
PBB-80	0.670/carcinogenic	0.805/carcinogenic	0.559/carcinogenic	0.712/carcinogenic	0.241/non		0.258/non		0.330/carcinogenic	0.499/single	0.374/carcinogenic	0.537/single	0.604/non
PBB-36	0.599/carcinogenic	0.777/carcinogenic	0.534/carcinogenic	0.724/carcinogenic	0.231/non		0.243/non		0.298/carcinogenic	0.510/single	0.364/carcinogenic	0.498/single	0.588/non
PBB-14	0.676/carcinogenic	0.760/carcinogenic	0.533/carcinogenic	0.696/carcinogenic	0.240/non		0.265/carcinogenic	0.173/multi	0.262/non		0.334/non		0.669/non
PBB-2	0.600/carcinogenic	0.736/carcinogenic	0.505/non	0.696/carcinogenic	0.228/non		0.268/carcinogenic	0.162/single	0.258/non		0.327/non		0.659/non
Biphenyl	0.607/carcinogenic	0.603/carcinogenic	0.540/carcinogenic	0.655/carcinogenic	0.270/carcinogenic	0.441/multi	0.315/carcinogenic	0.192/multi	0.244/non		0.325/non		0.767/mutagenic
PBB-80-B1	0.759/carcinogenic	0.802/carcinogenic	0.551/carcinogenic	0.766/carcinogenic	0.255/carcinogenic	0.419/multi	0.295/carcinogenic	0.166/single	0.376/carcinogenic	0.461/single	0.440/carcinogenic	0.560/single	0.680/non
PBB-11	0.587/non	0.716/carcinogenic	0.506/non	0.686/carcinogenic	0.233/non		0.255/non		0.298/carcinogenic	0.523/multi	0.368/carcinogenic	0.509/single	0.657/non

2, 2'-OH-PBB-80	0.662/carcinogenic	0.681/carcinogenic	0.494/non	0.764/carcinogenic	0.237/non		0.245/non		0.302/carcinogenic	0.523/multi	0.353/carcinogenic	0.556/single	0.400/non
PBB-153	0.669/carcinogenic	0.816/carcinogenic	0.555/carcinogenic	0.711/carcinogenic	0.241/non		0.257/non		0.337/carcinogenic	0.466/single	0.378/carcinogenic	0.536/single	0.597/non
PBB-118	0.685/carcinogenic	0.805/carcinogenic	0.548/carcinogenic	0.758/carcinogenic	0.220/non		0.232/non		0.314/carcinogenic	0.513/multi	0.366/carcinogenic	0.525/single	0.577/non
PBB-101	0.711/carcinogenic	0.849/carcinogenic	0.524/non	0.736/carcinogenic	0.239/non		0.241/non		0.337/carcinogenic	0.493/single	0.378/carcinogenic	0.543/single	0.512/non
PBB-99	0.692/carcinogenic	0.833/carcinogenic	0.523/non	0.751/carcinogenic	0.227/non		0.210/non		0.344/carcinogenic	0.508/single	0.370/carcinogenic	0.532/single	0.487/non
PBB-77	0.621/carcinogenic	0.756/carcinogenic	0.537/carcinogenic	0.753/carcinogenic	0.227/non		0.273/carcinogenic	0.172/multi	0.324/carcinogenic	0.513/multi	0.366/carcinogenic	0.525/single	0.662/non
PBB-70	0.706/carcinogenic	0.815/carcinogenic	0.534/carcinogenic	0.769/carcinogenic	0.220/non		0.247/non		0.314/carcinogenic	0.513/multi	0.366/carcinogenic	0.525/single	0.571/non
PBB-52	0.691/carcinogenic	0.808/carcinogenic	0.524/non	0.724/carcinogenic	0.239/non		0.265/carcinogenic	0.179/multi	0.337/carcinogenic	0.493/single	0.378/carcinogenic	0.543/single	0.586/non
PBB-49	0.692/carcinogenic	0.833/carcinogenic	0.526/carcinogenic	0.751/carcinogenic	0.227/non		0.210/non		0.344/carcinogenic	0.508/single	0.370/carcinogenic	0.532/single	0.502/non
PBB-37	0.643/carcinogenic	0.777/carcinogenic	0.507/non	0.764/carcinogenic	0.230/non		0.269/carcinogenic	0.160/single	0.319/carcinogenic	0.512/single	0.372/carcinogenic	0.506/single	0.594/non
PBB-12	0.628/carcinogenic	0.703/carcinogenic	0.499/non	0.738/carcinogenic	0.228/non		0.283/carcinogenic	0.166/single	0.258/non		0.334/non		0.695/non
PBB-3	0.695/carcinogenic	0.713/carcinogenic	0.486/non	0.719/carcinogenic	0.227/non		0.255/non		0.262/non		0.327/non		0.649/non
Biphenyl	0.607/carcinogenic	0.603/carcinogenic	0.540/carcinogenic	0.655/carcinogenic	0.270/carcinogenic	0.441/multi	0.315/carcinogenic	0.192/multi	0.244/non		0.325/non		0.767/mutagenic

PBDF	0.775/carcinogenic	0.820/carcinogenic	0.569/carcinogenic	0.763/carcinogenic	0.272/carcinogenic	0.408/multi	0.301/carcinogenic	0.165/single	0.406/carcinogenic	0.451/single	0.454/carcinogenic	0.557/single	0.707/non
PBB-47	0.670/carcinogenic	0.797/carcinogenic	0.517/non	0.739/carcinogenic	0.227/non		0.344069		0.344/carcinogenic	0.508/single	0.370/carcinogenic	0.532/single	0.535/non
PBB-17	0.697/carcinogenic	0.793/carcinogenic	0.487/non	0.766/carcinogenic	0.221/non		0.332623		0.333/carcinogenic	0.506/single	0.356/carcinogenic	0.507/single	0.525/non
PBB-18	0.716/carcinogenic	0.803/carcinogenic	0.503/non	0.752/carcinogenic	0.232/non		0.325914		0.326/carcinogenic	0.506/single	0.356/carcinogenic	0.507/single	0.607/non
PBB-4	0.653/carcinogenic	0.734/carcinogenic	0.490/non	0.703/carcinogenic	0.235/non		0.260/non		0.328/carcinogenic	0.520/multi	0.360/carcinogenic	0.517/single	0.669/non
PBB-1	0.754/carcinogenic	0.713/carcinogenic	0.511/non	0.708/carcinogenic	0.220/non		0.228/non		0.286/carcinogenic	0.522/multi	0.327/non		0.633/non
PBB-153-D1	0.617/carcinogenic	0.802/carcinogenic	0.535/carcinogenic	0.765/carcinogenic	0.230/non		0.241/non		0.310/carcinogenic	0.488/single	0.371/carcinogenic	0.549/single	0.527/non
PBB-153-D2	0.600/carcinogenic	0.677/carcinogenic	0.512/non	0.750/carcinogenic	0.234/non		0.249/non		0.298/carcinogenic	0.509/single	0.363/carcinogenic	0.542/single	0.572/non
D1	0.577/non	0.728/carcinogenic	0.545/carcinogenic	0.630/carcinogenic	0.220/non		0.217/non		0.294/carcinogenic	0.472/single	0.348/non		0.623/non
D1-A1	0.575/non	0.652/carcinogenic	0.526/carcinogenic	0.655/carcinogenic	0.208/non		0.230/non		0.277/carcinogenic	0.496/single	0.327/non		0.688/non
D1-A2	0.554/non	0.662/carcinogenic	0.531/carcinogenic	0.640/carcinogenic	0.213/non		0.251/non		0.241/non		0.315/non		0.746/mutagenic
D1-B1	0.594/non	0.694/carcinogenic	0.541/carcinogenic	0.671/carcinogenic	0.243/non		0.286/carcinogenic	0.150/single	0.347/carcinogenic	0.388/single	0.419/carcinogenic	0.563/single	0.735/mutagenic
D1-C1	0.645/carcinogenic	0.780/carcinogenic	0.434/non	0.639/carcinogenic	0.216/non		0.210/non		0.274/carcinogenic	0.491/single	0.304/non		0.563/non

D1-D1	0.569/non	0.781/carcinogenic	0.520/non	0.674/carcinogenic	0.211/non		0.206/non		0.267/carcinogenic	0.460/single	0.332/non		0.594/non
D1-D2	0.560/non	0.693/carcinogenic	0.511/non	0.661/carcinogenic	0.213/non		0.215/non		0.278/carcinogenic	0.471/single	0.337/non		0.616/non
D2	0.599/carcinogenic	0.757/carcinogenic	0.608/carcinogenic	0.732/carcinogenic	0.230/non		0.197/non		0.262/non		0.359/carcinogenic	0.524/single	0.605/non
D2-A1	0.599/carcinogenic	0.756/carcinogenic	0.627/carcinogenic	0.776/carcinogenic	0.213/non		0.188/non		0.240/non		0.343/non		0.620/non
D2-A2	0.567/non	0.603/carcinogenic	0.617/carcinogenic	0.737/carcinogenic	0.236/non		0.240/non		0.194/non		0.294/non		0.774/mutagenic
D2-B1	0.711/carcinogenic	0.774/carcinogenic	0.620/carcinogenic	0.801/carcinogenic	0.265/carcinogenic	0.267/single	0.219/non		0.297/carcinogenic	0.448/single	0.414/carcinogenic	0.535/single	0.731/mutagenic
D2-C1	0.664/carcinogenic	0.755/carcinogenic	0.552/carcinogenic	0.770/carcinogenic	0.240/non		0.232/non		0.292/carcinogenic	0.524/multi	0.348/non		0.730/mutagenic
D2-D1	0.584/non	0.762/carcinogenic	0.577/carcinogenic	0.766/carcinogenic	0.220/non		0.188/non		0.230/non		0.334/non		0.579/non
D2-D2	0.579/non	0.698/carcinogenic	0.563/carcinogenic	0.782/carcinogenic	0.220/non		0.198/non		0.225/non		0.304/non		0.562/non
D3	0.593/non	0.701/carcinogenic	0.555/carcinogenic	0.768/carcinogenic	0.249/carcinogenic	0.347/multi	0.234/non		0.347/carcinogenic	0.524/multi	0.373/carcinogenic	0.563/single	0.661/non
D3-A1	0.593/non	0.687/carcinogenic	0.555/carcinogenic	0.826/carcinogenic	0.223/non		0.205/non		0.329/carcinogenic	0.532/multi	0.367/carcinogenic	0.555/single	0.645/non
D3-A2	0.704/carcinogenic	0.706/carcinogenic	0.571/carcinogenic	0.743/carcinogenic	0.230/non		0.209/non		0.269/carcinogenic	0.532/multi	0.312/non		0.716/non
D3-A3	0.577/non	0.608/carcinogenic	0.509/non	0.767/carcinogenic	0.225/non		0.265/carcinogenic	0.149/single	0.247/non		0.304/non		0.693/non

D3-B1	0.629/carcinogenic	0.735/carcinogenic	0.568/carcinogenic	0.804/carcinogenic	0.262/carcinogenic	0.333/single	0.258/non		0.377/carcinogenic	0.471/single	0.420/carcinogenic	0.562/single	0.730/mutagenic
D3-C1	0.664/carcinogenic	0.755/carcinogenic	0.552/carcinogenic	0.770/carcinogenic	0.240/non		0.232/non		0.292/carcinogenic	0.524/multi	0.348/non		0.730/mutagenic
D3-C2	0.581/non	0.614/carcinogenic	0.488/non	0.645/carcinogenic	0.232/non		0.266/carcinogenic	0.151/single	0.324/carcinogenic	0.517/multi	0.333/non		0.618/non
D3-D1	0.559/non	0.721/carcinogenic	0.544/carcinogenic	0.795/carcinogenic	0.231/non		0.218/non		0.307/carcinogenic	0.526/multi	0.363/carcinogenic	0.564/single	0.648/non
D3-D2	0.528/non	0.633/carcinogenic	0.519/non	0.788/carcinogenic	0.235/non		0.221/non		0.327/carcinogenic	0.528/multi	0.333/non		0.668/non

Table S2 Biotoxicity evaluation of PBBs and their substitutes' transformation products

PBBs and their transformation products	Oral LD in rats$_{50}$ (g/kg)	Toxicity classification	Maximum tolerated dose in rats - feed/water (g/kg)	Toxicity classification	Maximum tolerated dose in rats - gavage (g/kg)	Toxicity classification	Long-term oral minimum level of toxicity and side effects in rats (g/kg)	Toxicity classification	Rat inhalation LC$_{50}$ (mg/m3/h)	Toxicity classification	Blackhead dullfish toxicity LC$_{50}$ (g/l)	Hazard classification	Daphnia magna toxicity EC$_{50}$ (mg/l)	Hazard classification
PBB-3	1.559	+	0.061	++	0.017	+++	0.049	+++	5490.250	+++	7.98E-04	**Extremely high**	0.114	**Extremely high**
Biphenyl	1.299	+	0.060	++	0.173	++	0.082	++	6954.960	+++	2.01E-03	High	0.287	**Extremely high**
PBB-3-B1	0.662	+	0.051	++	0.020	+++	0.029	+++	12030.700	++	6.02E-04	**Extremely high**	0.244	**Extremely high**
4'-OH-PBB-3	0.916	+	0.207	++	0.009	+++	0.052	++	3290.690	+++	8.93E-04	**Extremely high**	0.518	**Extremely high**
4'-Br-3-MeO-4-OH-biphenyl	1.770	+	0.132	++	4.51E-04	++++	0.093	++	8404.590	++	1.60E-03	High	1.167	High
PBB-4	3.160	−	0.036	+++	0.013	+++	0.085	++	3837.900	+++	1.73E−04	**Extremely high**	0.065	**Extremely high**
PBB−1	2.004	−	0.061	++	0.017	+++	0.068	++	5490.250	+++	7.98E-04	**Extremely high**	0.142	**Extremely high**

Biphenyl	1.299	+	0.060	++	0.173	++	0.082	++	6954.960	+++	2.01E-03	High	0.287	**Extremely high**
4-MBDF	0.401	+	0.051	++	0.020	+++	0.023	+++	7484.790	+++	6.02E-04	**Extremely high**	0.257	**Extremely high**
DF	0.628	+	0.051	++	0.202	++	0.035	+++	9756.150	++	1.56E-03	High	0.702	**Extremely high**
PBB-4-D1	0.758	+	0.121	++	0.007	+++	0.104	++	2147.970	+++	1.91E-04	**Extremely high**	0.191	**Extremely high**
PBB-4-D2	0.989	+	0.184	++	0.003	++++	0.125	++	1275.360	++++	3.47E-04	**Extremely high**	0.215	**Extremely high**
PBB-15	1.901	+	0.036	+++	0.013	+++	0.033	+++	4043.370	+++	1.73E-04	**Extremely high**	0.068	**Extremely high**
PBB-3	1.559	+	0.061	++	0.017	+++	0.049	+++	5490.250	+++	7.98E-04	**Extremely high**	0.114	**Extremely high**
Biphenyl	1.299	+	0.060	++	0.173	++	0.082	++	6954.960	+++	2.01E-03	High	0.287	**Extremely high**
PBB-15-B1	0.464	+	0.030	+++	0.015	+++	0.016	+++	8733.360	++	1.29E-04	**Extremely high**	0.138	**Extremely high**
4-OH-PBB-13	1.311	+	0.121	++	0.007	+++	0.073	++	3637.370	+++	1.91E-04	**Extremely high**	0.259	**Extremely high**
3-OH-PBB-15	0.820	+	0.121	++	0.007	+++	0.067	++	3637.370	+++	1.91E-04	**Extremely high**	0.288	**Extremely high**
4-Br-3-MeO-4'-OH-biphenyl	2.373	−	0.132	++	4.51E-04	++++	0.084	++	8404.590	++	1.60E-03	High	0.940	**Extremely high**
PBB-29	1.609	+	0.020	+++	0.010	+++	0.082	++	4036.130	+++	3.54E-05	**Extremely high**	0.022	**Extremely high**
PBB-9	2.822	−	0.036	+++	0.013	+++	0.077	++	6168.850	+++	1.73E-04	**Extremely high**	0.062	**Extremely high**
PBB-7	3.714	−	0.036	+++	0.013	+++	0.077	++	6168.850	+++	1.73E-04	**Extremely high**	0.063	**Extremely high**
PBB-3	1.559	+	0.061	++	0.017	+++	0.049	+++	5490.250	+++	7.98E-04	**Extremely high**	0.114	**Extremely high**
PBB-2	1.462	+	0.061	++	0.017	+++	0.047	+++	8824.760	++	7.98E-04	**Extremely high**	0.101	**Extremely high**
PBB-1	2.004	−	0.061	++	0.017	+++	0.068	++	5490.250	+++	7.98E-04	**Extremely high**	0.142	**Extremely high**
Biphenyl	1.299	+	0.060	++	0.173	++	0.082	++	6954.960	+++	2.01E-03	High	0.287	**Extremely high**
PBB-29-B1	0.350	+	0.030	+++	0.015	+++	0.042	+++	8289.560	++	1.29E-04	**Extremely high**	0.129	**Extremely high**
PBB-29-D1	0.707	+	0.067	++	0.005	+++	0.136	++	2236.670	+++	3.85E-05	**Extremely high**	0.062	**Extremely high**
PBB-29-D2	1.063	+	0.101	++	0.002	++++	0.235	++	1316.060	++++	6.94E-05	**Extremely high**	0.058	**Extremely high**
PBB-80	1.528	+	0.011	+++	0.007	+++	0.029	+++	3579.690	+++	6.90E-06	**Extremely high**	0.010	**Extremely high**
PBB-36	1.836	+	0.020	+++	0.010	+++	0.037	+++	4036.140	+++	3.54E-05	**Extremely high**	0.021	**Extremely high**
PBB-14	2.071	−	0.036	+++	0.013	+++	0.061	++	6168.850	+++	1.73E-04	**Extremely high**	0.042	**Extremely high**

PBB-2	1.462	+	0.061	++	0.017	+++	0.047	+++	8824.760	++	7.98E-04	**Extremely high**	0.101	**Extremely high**
Biphenyl	1.299	+	0.060	++	0.173	++	0.082	++	6954.960	+++	2.01E-03	High	0.287	**Extremely high**
PBB-80-B1	0.443	+	0.009	+++	0.007	+++	0.015	+++	4741.000	+++	5.06E-06	**Extremely high**	0.026	**Extremely high**
PBB-11	2.199	−	0.036	+++	0.013	+++	0.043	+++	6168.850	+++	1.73E-04	**Extremely high**	0.056	**Extremely high**
2, 2'-OH-PBB-80	0.967	+	0.053	++	0.001	++++	0.098	++	1152.390	++++	1.34E-05	**Extremely high**	0.046	**Extremely high**
PBB-153	0.963	+	0.003	++++	0.003	++++	0.032	+++	1304.130	++++	2.44E-07	**Extremely high**	0.002	**Extremely high**
PBB-118	1.308	+	0.006	+++	0.004	++++	0.036	+++	1627.910	++++	1.31E-06	**Extremely high**	0.004	**Extremely high**
PBB-101	1.308	+	0.006	+++	0.004	++++	0.037	+++	1627.910	++++	1.31E-06	**Extremely high**	0.005	**Extremely high**
PBB-99	1.308	+	0.006	+++	0.004	++++	0.037	+++	1627.910	++++	1.31E-06	**Extremely high**	0.005	**Extremely high**
PBB-77	1.363	+	0.011	+++	0.007	+++	0.042	+++	2669.050	+++	6.90E-06	**Extremely high**	0.012	**Extremely high**
PBB-70	1.680	+	0.011	+++	0.007	+++	0.045	+++	2669.050	+++	6.90E-06	**Extremely high**	0.014	**Extremely high**
PBB-52	1.961	+	0.011	+++	0.007	+++	0.040	+++	2669.050	+++	6.90E-06	**Extremely high**	0.012	**Extremely high**
PBB-49	2.581	−	0.011	+++	0.007	+++	0.040	+++	2669.050	+++	6.90E-06	**Extremely high**	0.012	**Extremely high**
PBB-37	1.491	+	0.020	+++	0.010	+++	0.045	+++	4252.220	+++	3.54E-05	**Extremely high**	0.035	**Extremely high**
PBB-12	1.306	+	0.036	+++	0.013	+++	0.071	++	6168.850	+++	1.73E-04	**Extremely high**	0.053	**Extremely high**
PBB-3	1.559	+	0.061	++	0.017	+++	0.049	+++	5490.250	+++	7.98E-04	**Extremely high**	0.114	**Extremely high**
Biphenyl	1.299	+	0.060	++	0.173	++	0.082	++	6954.960	+++	2.01E-03	High	0.287	**Extremely high**
PBDF	0.259	++	0.004	++++	0.005	+++	0.017	+++	2879.620	+++	9.57E-07	**Extremely high**	0.012	**Extremely high**
PBB-47	2.581	−	0.011	+++	0.007	+++	0.040	+++	2,669.05	+++	6.90E-06	**Extremely high**	0.012	**Extremely high**
PBB-17	4.932	−	0.020	+++	0.010	+++	0.090	++	4036.130	+++	3.54E-05	**Extremely high**	0.027	**Extremely high**
PBB-18	3.747	−	0.020	+++	0.010	+++	0.090	++	4036.130	+++	3.54E-05	**Extremely high**	0.027	**Extremely high**
PBB-4	3.160	−	0.036	+++	0.013	+++	0.085	++	3837.900	+++	0.0002	**Extremely high**	0.065	**Extremely high**
PBB-1	2.004	−	0.061	++	0.017	+++	0.068	++	5490.250	+++	7.98E-04	**Extremely high**	0.142	**Extremely high**
PBB-153-D1	0.283	++	0.009	+++	0.001	++++	0.045	+++	711.981	++++	2.62E-07	**Extremely high**	0.006	**Extremely high**
PBB-153-D2	0.340	+	0.014	+++	0.001	++++	0.072	++	413.101	++++	4.65E-07	**Extremely high**	0.006	**Extremely high**
D1	1.578	+	0.013	+++	0.018	+++	0.022	+++	3025.050	+++	1.67E-06	**Extremely high**	0.029	**Extremely high**

D1-A1	1.606	+	0.026	+++	0.026	+++	0.024	+++	3722.180	+++	8.84E-06	**Extremely high**	0.063	**Extremely high**
D1-A2	2.044	−	0.081	++	0.020	+++	0.049	+++	2386.710	+++	6.16E-04	**Extremely high**	0.405	**Extremely high**
D1-B1	0.358	+	0.021	+++	0.022	+++	0.008	+++	5675.340	+++	8.91E-06	**Extremely high**	0.102	**Extremely high**
D1-C1	3.951	−	0.051	++	0.181	++	0.113	++	26697.100	++	1.36E-04	**Extremely high**	0.327	**Extremely high**
D1-D1	0.559	+	0.043	+++	0.007	+++	0.021	+++	1908.480	++++	2.47E-06	**Extremely high**	0.060	**Extremely high**
D1-D2	0.505	+	0.064	++	0.003	++++	0.030	+++	1109.920	++++	4.40E-06	**Extremely high**	0.051	**Extremely high**
D2	1.367	+	0.056	++	1.73E-04	++++	0.091	++	333.184	++++	7.83E-06	**Extremely high**	0.181	**Extremely high**
D2-A1	1.421	+	0.108	++	2.57E-04	++++	0.139	++	406.525	++++	4.12E-05	**Extremely high**	0.540	**Extremely high**
D2-A2	2.360	−	0.399	+	0.006	+++	0.284	++	1326.910	++++	2.90E-03	High	7.633	High
D2-B1	0.337	+	0.087	++	2.14E-04	++++	0.039	+++	832.718	++++	4.16E-05	**Extremely high**	0.742	**Extremely high**
D2-C1	1.016	+	0.041	+++	1.56E-04	++++	0.121	++	1358.040	++++	1.11E-04	**Extremely high**	0.049	**Extremely high**
D2-D1	0.469	+	0.112	++	6.89E-05	++++	0.090	++	194.189	++++	1.40E-05	**Extremely high**	0.160	**Extremely high**
D2-D2	0.460	+	0.168	++	2.75E-05	++++	0.112	++	113.086	++++	2.49E-05	**Extremely high**	0.147	**Extremely high**
D3	0.657	+	0.009	+++	2.77E-05	++++	0.047	+++	224.435	++++	5.83E-06	**Extremely high**	0.005	**Extremely high**
D3-A1	0.872	+	0.018	+++	4.12E-05	++++	0.052	++	273.605	++++	3.06E-05	**Extremely high**	0.010	**Extremely high**
D3-A2	1.299	+	0.041	+++	1.56E-04	++++	0.109	++	1358.040	++++	1.11E-04	**Extremely high**	0.055	**Extremely high**
D3-A3	1.540	+	0.053	++	0.001	++++	0.084	++	2438.660	+++	1.47E-03	High	0.057	**Extremely high**
D3-B1	0.193	++	0.014	+++	3.44E-05	++++	0.022	+++	560.553	++++	3.09E-05	**Extremely high**	0.022	**Extremely high**
D3-C1	1.016	+	0.041	+++	1.56E-04	++++	0.121	++	1358.040	++++	1.11E-04	**Extremely high**	0.049	**Extremely high**
D3-C2	2.124	−	0.030	+++	4.44E-04	++++	0.088	++	1471.920	++++	3.07E-04	**Extremely high**	0.014	**Extremely high**
D3-D1	0.365	+	0.030	+++	1.07E-05	++++	0.063	++	141.817	++++	8.66E-06	**Extremely high**	0.012	**Extremely high**
D3-D2	0.428	+	0.045	+++	4.28E-06	++++	0.094	++	82.599	++++	1.54E-05	**Extremely high**	0.012	**Extremely high**

Table S3 Biological tissue toxicity of PBBs and their substitutes' transformation products and evaluation of potential developmental toxicity

PBBs and their transformation products	Potential developmental type	Skin Non-/Irritation	Mild/moderate skin irritation	Skin non-/ sensitization	Weak/strong allergenicity	Eye non-/irritant	Mild/moderate-severe eye irritation	Moderate/severe eye irritation
PBB-3	0.497/non	0.971/non		0.850/sensitization	0.975/strong	0.975/irritation	0.731/mild	
Biphenyl	0.439/non	0.976/ irritation	0.225/mild	0.807/sensitization	0.917/strong	0.971/non		
PBB-3-B1	0.594/toxic	0.975/irritation	0.055/mild	0.699/non		1.000/irritation	0.770/mild	
4'-OH-PBB-3	0.598/toxic	0.964/non		0.803/sensitization	0.982/strong	1.000/irritation	0.811/mild	
4'-Br-3-MeO-4-OH-biphenyl	0.672/toxic	0.967/non		0.818/sensitization	0.991/strong	1.000/irritation	0.746/mild	
PBB-4	0.513/non	0.975/irritation	0.123/mild	0.831/sensitization	0.923/strong	1.000/irritation	0.771/mild	
PBB-1	0.477/non	0.973/irritation	0.083/mild	0.842/sensitization	0.970/strong	1.000/irritation	0.758/mild	
Biphenyl	0.439/non	0.976/irritation	0.225/mild	0.807/sensitization	0.917/strong	0.971/non		
4-MBDF	0.582/toxic	0.975/irritation	0.074/mild	0.699/non-sensitization		1.000/irritation	0.721/mild	
DF	0.538/toxic	0.978/irritation	0.221/mild	0.649/non-sensitization		1.000/irritation	0.763/mild	
PBB-4-D1	0.484/non	0.970/non		0.801/sensitization	0.972/strong	1.000/irritation	0.813/mild	
PBB-4-D2	0.513/non	0.966/non		0.787/sensitization	0.980/strong	1.000/irritation	0.826/moderate - severe	0.676/single
PBB-15	0.562/toxic	0.973/irritation	0.058/mild	0.839/sensitization	0.925/strong	1.000/irritation	0.747/mild	
PBB-3	0.497/non	0.971/non		0.850/sensitization	0.975/strong	0.975/irritation	0.731/mild	
Biphenyl	0.439/non	0.976/irritation	0.225/mild	0.807/sensitization	0.917/strong	0.971/non		
PBB-15-B1	0.573/toxic	0.977/irritation	0.093/mild	0.753/sensitization	0.978/strong	1.000/irritation	0.780/mild	
4-OH-PBB-13	0.599/toxic	0.969/non		0.810/sensitization	0.974/strong	1.000/irritation	0.830/moderate - severe	0.688/multi
3-OH-PBB-15	0.570/toxic	0.967/non		0.822/sensitization	0.971/strong	1.000/irritation	0.782/mild	
4-Br-3-MeO-4'-OH-biphenyl	0.624/toxic	0.967/non		0.802/sensitization	0.988/strong	1.000/irritation	0.734/mild	
PBB-29	0.516/non	0.977/irritation	0.074/mild	0.822/sensitization	0.926/strong	0.975/irritation	0.769/mild	
PBB-9	0.519/non	0.977/irritation	0.046/mild	0.838/sensitization	0.929/strong	0.976/irritation	0.779/mild	
PBB-7	0.525/non	0.976/irritation	0.046/mild	0.854/sensitization	0.974/strong	1.000/irritation	0.766/mild	
PBB-3	0.497/non	0.971/non		0.850/sensitization	0.975/strong	0.975/irritation	0.731/mild	
PBB-2	0.497/non	0.974/irritation	0.037/mild	0.839/sensitization	0.975/strong	1.000/irritation	0.749/mild	

PBB-1	0.477/non	0.973/irritation	0.083/mild	0.842/sensitization	0.970/strong	1.000/irritation	0.758/mild	
Biphenyl	0.439/non	0.976/irritation	0.225/mild	0.807/sensitization	0.917/strong	0.971/non		
PBB-29-B1	0.581/toxic	0.978/irritation	0.086/mild	0.688/non-sensitization		1.000/irritation	0.762/mild	
PBB-29-D1	0.519/non	0.974/irritation	0.124/mild	0.758/sensitization	0.977/strong	1.000/irritation	0.821/moderate - severe	0.721/multi
PBB-29-D2	0.522/non	0.967/non		0.757/sensitization	0.980/strong	1.000/irritation	0.824/moderate - severe	0.710/multi
PBB-80	0.604/toxic	0.978/irritation	0.126/mild	0.814/sensitization	0.921/strong	0.975/irritation	0.805/mild	
PBB-36	0.561/toxic	0.977/irritation	0.043/mild	0.816/sensitization	0.971/strong	0.976/irritation	0.792/mild	
PBB-14	0.545/toxic	0.976/irritation	0.060/mild	0.843/sensitization	0.928/strong	0.976/irritation	0.758/mild	
PBB-2	0.497/non	0.974/irritation	0.037/mild	0.839/sensitization	0.975/strong	1.000/irritation	0.749/mild	
Biphenyl	0.439/non	0.976/irritation	0.225/mild	0.807/sensitization	0.917/strong	0.971/non		
PBB-80-B1	0.601/toxic	0.978/irritation	0.126/mild	0.774/sensitization	0.974/strong	1.000/irritation	0.787/mild	
PBB-11	0.542/toxic	0.977/irritation	0.047/mild	0.829/sensitization	0.928/strong	1.000/irritation	0.786/mild	
2, 2'-OH-PBB-80	0.713/toxic	0.962/non		0.788/sensitization	0.975/strong	1.000/irritation	0.837/moderate - severe	0.666/single
PBB-153	0.581/toxic	0.978/irritation	0.166/mild	0.814/sensitization	0.918/strong	0.976/irritation	0.810/mild	
PBB-118	0.568/toxic	0.977/irritation	0.066/mild	0.818/sensitization	0.922/strong	1.000/irritation	0.788/mild	
PBB-101	0.593/toxic	0.978/irritation	0.086/mild	0.818/sensitization	0.925/strong	1.000/irritation	0.820/moderate - severe	0.637/single
PBB-99	0.581/toxic	0.977/irritation	0.069/mild	0.827/sensitization	0.927/strong	1.000/irritation	0.798/mild	
PBB-77	0.551/toxic	0.978/irritation	0.071/mild	0.809/sensitization	0.919/strong	0.976/irritation	0.792/mild	
PBB-70	0.564/toxic	0.978/irritation	0.053/mild	0.809/sensitization	0.922/strong	0.999/irritation	0.800/mild	
PBB-52	0.576/toxic	0.978/irritation	0.114/mild	0.809/sensitization	0.922/strong	0.976/irritation	0.816/moderate - severe	0.637/single
PBB-49	0.581/toxic	0.977/irritation	0.071/mild	0.827/sensitization	0.927/strong	0.976/irritation	0.802/mild	
PBB-37	0.565/toxic	0.976/irritation	0.031/mild	0.818/sensitization	0.925/strong	0.976/irritation	0.777/mild	
PBB-12	0.507/non	0.977/irritation	0.035/mild	0.838/sensitization	0.926/strong	1.000/irritation	0.769/mild	
PBB-3	0.497/non	0.971/non		0.850/sensitization	0.975/strong	0.975/irritation	0.731/mild	
Biphenyl	0.439/non	0.976/irritation	0.225/mild	0.807/sensitization	0.917/strong	0.971/non		
PBDF	0.599/toxic	0.995/irritation	0.152/mild	0.749/sensitization	0.972/strong	1.000/irritation	0.763/mild	

PBB-47	0.570/toxic	0.976/irritation	0.094/mild	0.827/sensitization	0.927/strong	0.999/irritation	0.792/mild	
PBB-17	0.534/non	0.975/irritation	0.073/mild	0.827/sensitization	0.971/strong	1.000/irritation	0.782/mild	
PBB-18	0.527/non	0.976/irritation	0.072/mild	0.809/sensitization	0.926/strong	0.976/irritation	0.793/mild	
PBB-4	0.513/non	0.975/irritation	0.123/mild	0.831/sensitization	0.923/strong	1.000/irritation	0.771/mild	
PBB-1	0.477/non	0.973/irritation	0.083/mild	0.842/sensitization	0.970/strong	1.000/irritation	0.758/mild	
PBB-153-D1	0.611/toxic	0.976/irritation	0.139/mild	0.789/sensitization	0.927/strong	1.000/irritation	0.829/moderate - severe	0.667/single
PBB-153-D2	0.612/toxic	0.971/non		0.788/sensitization	0.972/strong	1.000/irritation	0.829/moderate - severe	0.662/single
D1	0.599/toxic	0.974/irritation	0.023/mild	0.816/sensitization	0.977/strong	1.000/irritation	0.737/mild	
D1-A1	0.569/toxic	0.973/non		0.811/sensitization	0.978/strong	1.000/irritation	0.692/mild	
D1-A2	0.509/non	0.967/non		0.827/sensitization	0.984/strong	0.976/irritation	0.651/mild	
D1-B1	0.558/toxic	0.977/irritation	0.015/mild	0.729/non-sensitization		1.000/irritation	0.755/mild	
D1-C1	0.538/toxic	0.973/non		0.849/sensitization	0.974/strong	1.000/irritation	0.764/mild	
D1-D1	0.631/toxic	0.968/non		0.773/sensitization	0.982/strong	1.000/irritation	0.782/mild	
D1-D2	0.612/toxic	0.968/non		0.788/sensitization	0.979/strong	1.000/irritation	0.812/mild	
D2	0.560/toxic	0.603/non		0.762/sensitization	0.978/strong	1.000/irritation	0.822/moderate - severe	0.514/single
D2-A1	0.563/toxic	0.435/non		0.774/sensitization	0.979/strong	1.000/irritation	0.779/mild	
D2-A2	0.431/non	0.321/non		0.786/sensitization	0.978/strong	1.000/irritation	0.725/mild	
D2-B1	0.590/toxic	0.681/non		0.717/non-sensitization		1.000/irritation	0.810/mild	
D2-C1	0.497/non	0.815/non		0.823/sensitization	0.979/strong	1.000/irritation	0.771/mild	
D2-D1	0.592/toxic	0.463/non		0.789/sensitization	0.985/strong	1.000/irritation	0.835/moderate - severe	0.497/single
D2-D2	0.574/toxic	0.457/non		0.800/sensitization	0.984/strong	1.000/irritation	0.837/moderate - severe	0.470/single
D3	0.606/toxic	0.916/non		0.852/sensitization	0.929/strong	0.975/irritation	0.789/mild	
D3-A1	0.595/toxic	0.900/non		0.856/sensitization	0.973/strong	0.976/irritation	0.718/mild	
D3-A2	0.504/non	0.923/non		0.841/sensitization	0.978/strong	1.000/irritation	0.751/mild	
D3-A3	0.515/non	0.973/non		0.883/sensitization	0.978/strong	0.975/irritation	0.657/mild	
D3-B1	0.651/toxic	0.931/non		0.807/sensitization	0.978/strong	1.000/irritation	0.749/mild	

D3-C1	0.497/non	0.815/non		0.823/sensitization	0.979/strong	1.000/irritation	0.771/mild	
D3-C2	0.545/toxic	0.953/non		0.879/sensitization	0.979/strong	0.974/non		
D3-D1	0.621/toxic	0.896/non		0.840/sensitization	0.981/strong	0.999/irritation	0.814/moderate - severe	0.462/single
D3-D2	0.606/toxic	0.844/non		0.847/sensitization	0.980/strong	0.976/irritation	0.818/moderate - severe	0.443/single
